# Phase II Feasibility Study of the Efficacy, Tolerability, and Impact on the Gut Microbiome of a Low-Residue (Fiber) Diet in Adult Patients With Mitochondrial Disease

**DOI:** 10.1016/j.gastha.2022.03.007

**Published:** 2022-07-01

**Authors:** David Houghton, Yi Shiau Ng, Matthew A. Jackson, Renae Stefanetti, Paula Hynd, Micheál Mac Aogáin, Christopher J. Stewart, Christopher A. Lamb, Alexandra Bright, Catherine Feeney, Jane Newman, Doug M. Turnbull, Robert McFarland, Alasdair P. Blain, Gráinne S. Gorman

**Affiliations:** 1 Faculty of Medical Science, Wellcome Centre for Mitochondrial Research, Translational and Clinical Research Institute, Newcastle University, Newcastle upon Tyne, UK; 2 NIHR Newcastle Biomedical Research Centre, Newcastle on Tyne, UK; 3 Kennedy Institute of Rheumatology, University of Oxford, Oxford, UK; 4 Lee Kong Chian School of Medicine, Nanyang Technological University, Novena, Singapore; 5 Biochemical Genetics Laboratory, Department of Biochemistry, St. James Hospital, Dublin, Ireland; 6 Faculty of Medical Science, Translational and Clinical Research Institute, Newcastle University, Newcastle upon Tyne, UK

**Keywords:** Gastrointestinal Dysmotility, Mitochondria, Diet, Residue, Gut Microbiome

## Abstract

**Background and Aims:**

Gastrointestinal (GI) dysmotility is a common and debilitating clinical manifestation in patients with mitochondrial DNA (mtDNA)–related disease with no curative and few effective symptomatic therapies. A low-residue diet (LRD) has been shown to be effective at reducing bowel urgency, pain, and distension in functional GI-related conditions. We assessed tolerability and effects of an LRD on bowel habits in patients with mtDNA-related disease.

**Methods:**

This was a 12-week single-arm pilot study in patients with genetically determined primary mtDNA-related disease, meeting the ROME III constipation criteria. The co-primary outcomes were tolerability of an LRD (<10 g fiber per day) assessed by food diaries and changes in stool frequency and consistency. The secondary outcomes included GI symptoms, disease burden, laxatives, physical activity levels, colonic transit time using radiopaque markers, gut microbiome (patients and controls), and metabolomics. The gut microbiome of the mtDNA-related disease patients was compared against controls for observational purpose only.

**Results:**

Twenty-eight patients were enrolled, and 24 completed the LRD intervention. The LRD was well tolerated with a mean fold change of −34% in dietary fiber (5.3 ± 10.4 grams) per day (*P* = .03, confidence interval = 0.7–9.9) with no adverse events. The proportion of stool samples with normal stool consistency increased from 36% to 49% (*P* = .01); GI symptoms and laxative use were reduced. However, the LRD did not change stool frequency, stool output, and colonic transit time. The gut microbiome was significantly different between patients and controls but was not modulated by the dietary intervention.

**Conclusion:**

The LRD in patients with mtDNA-related mitochondrial disease and significant constipation is well tolerated and a promising treatment for alleviating GI symptoms. These positive findings should be confirmed in a randomized controlled trial.

ClinicalTrials.gov Identifier: NCT03388528.

## Introduction

Mitochondrial diseases are a clinically diverse group of genetic disorders that are characterized by defects in oxidative phosphorylation caused by mutations in either the nuclear or the mitochondrial (mt) genome.^1^ Mitochondrial diseases are one of the most common groups of inherited neurometabolic disorders with mutations in mtDNA being the most common causative genetic defects in adult-related mitochondrial disease.^1^ The age of the onset is variable, and clinical expression of mtDNA-related diseases is wide ranging, but often results in significant morbidity and mortality.^1^ Gastrointestinal (GI) dysmotility is a frequent, debilitating manifestation and reported in up to 65% of patients with mtDNA-related disease,^2^^,^^3^ comparable with other common neurological disorders.^4^ The GI symptoms consistently include dysphagia, abdominal pain, abdominal distention, bacterial overgrowth, constipation, and, in severe cases, intestinal pseudo-obstruction,^2^ mimicking an acute surgical abdomen.^5^^,^^6^

Although the pathological mechanisms underlying the development of GI dysmotility and associated symptoms remain elusive, the potential factors include visceral myopathy^2^ and/or impaired coordination of intrinsic and extrinsic pathways of the GI tract.^7^ Furthermore, mitochondrial dysfunction of GI smooth muscle as demonstrated in mitochondrial neurogastrointestinal encephalomyopathy patients^5^ and in mice with mtDNA polymerase gamma mutation, *Polg*^*D257A*^,^8^ is likely to contribute to GI motility, a key determinant of gut microbiome composition,^9^ although to date, no research into the gut microbiome in mtDNA disease has been conducted. Although the etiology of the GI symptoms in mtDNA disease is likely to be multifactorial, insights from other disorders that share a clinical phenotype with mtDNA may provide further insight. For example, the gut microbiome has been implicated in the pathophysiology of various GI, metabolic, and neurological disorders.^10^ Indeed the gut microbiome is crucial for GI integrity, immunity, drug metabolism, nutrient digestion, and absorption^11^ and can facilitate gut motility, in part, through the synthesis of important neurotransmitters such as acetylcholine, essential for providing excitatory stimulation and smooth muscle contraction.^10^

Management of GI dysmotility in patients with mtDNA-related disease is complex and often personalized to each patient, incorporating optimization of nutrition, fluid intake, avoiding fasting, and remaining active (https://www.newcastle-mitochondria.com/wp-content/cache/all/clinical-professional-home-page/clinical-publications/clinical-guidelines/index.html). Alternatively, high-fiber diets are routinely implemented in bowel disorders, such as chronic idiopathic constipation^12^ and irritable bowel syndrome (IBS),^13^ although some evidence suggests that fiber can exacerbate GI symptoms including pain, distension, and urgency.^14^ A low-residue diet (LRD) is a form of low-fiber diet designed to minimize mechanical irritation caused by food residue and fiber, thereby reducing GI workload and the associated GI symptoms such as abdominal pain and distension.^15^ An LRD has been reported to be well tolerated and efficacious for preparing patients before/after bowel surgery.^15^ In addition, an LRD has been demonstrated to decrease bowel urgency, diarrhea, pain, and distension in IBS^16^ and idiopathic constipation^17^ and relieve GI symptoms in stricturing Crohn's disease.^18^ No trial of dietary manipulation in GI dysmotility associated with mtDNA disease has been conducted to date. We conducted a single-arm pilot study to test the tolerability and the effects of an LRD on bowel habits in mtDNA-related disease patients with GI dysmotility.

## Materials and Methods

### Participants

Patients were eligible for inclusion if they had a genetic confirmation of mitochondrial disease, aged ≥18 years, met the ROME III criteria of constipation (≤3 bowel movements/wk, hard or lumpy stools with straining, and sensation of obstruction and incomplete evacuations in at least 25% of bowel movements),^19^ as a measure of GI symptom severity, at least 3 months of a stable GI drug regimen prior to study inclusion, and no known hypersensitivities to any of the ingredients in the preparations and not already implementing an LRD (inclusion/exclusion criteria are further detailed in Supplemental Appendix for patients and controls). mtDNA disease presents with significant variability within and between genetic mutations. However, of the 24 patients included in this study, 20 harbored the 3243A > G mtDNA mutation, the most common cause of adult-onset mtDNA disease,^3^ and given the rarity of this disorder, this represents a good sample size. Furthermore, all patients included in this study shared the same GI symptoms and were selected based on meeting stringent criteria described here. All patients were prescribed one Forceval capsule, a multivitamin and mineral supplement, as part of routine care by a clinical dietician. Patients were advised to continue using laxatives as required and to keep records of their use. All participants provided informed written consent, and the study was approved and performed under the ethical guidelines issued by our institution and complied with the Declaration of Helsinki.

### Design and Procedures

**Figure 1:**
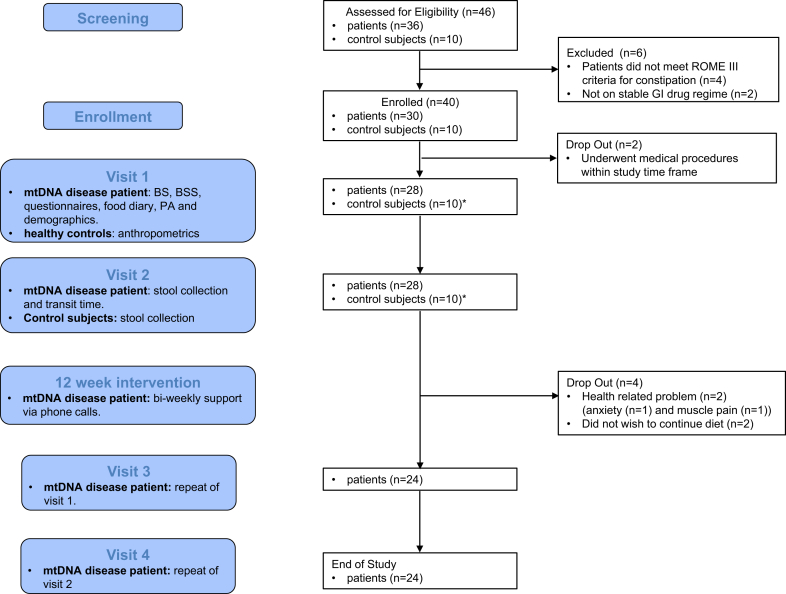
The consort diagram of the trial profile.

We conducted a single-arm pilot study recruiting from the NHS Highly Specialised Service for Rare Mitochondrial Disorders in Newcastle upon Tyne and the UK Mitochondrial Disease Patient Cohort (Ref: 13/NE/0326). The trial protocol was approved by the National Research Ethics Service Committee North East & Tyne and Wear South Research Ethics Committee (Ref: 17/NE/0193). The study was registered with ClinicalTrials.gov: NCT03388528. Eligible patients were invited to attend a baseline visit (visit 1, Figure 1) and enroll in the study (a full description of procedures is detailed in Supplemental Appendix).

### Intervention and Outcomes

The co-primary outcomes were tolerability of an LRD and stool frequency and consistency. Tolerability was assessed by food frequency diaries, and stool frequency and consistency were assessed using the Bristol stool score according to the ROME III criteria. The secondary outcomes included the following: colonic transit time (CTT) using retention of radiopaque markers (ROM); self-reported symptoms as measured by the Patient Assessment of Constipation-Symptoms (PAC-SYM) questionnaire and ROME III criteria, both assessments of GI symptom severity; anthropometrics; sleep duration and physical activity; gut metabolites; GI hormones and peptides; and gut microbiome, and dietary intake was recorded over 72 hours (1 weekend and 2 week days) using INTAKE24,^20^ an open-source self-completed computerized dietary recall system based on multiple-pass 24-hour recall that has been validated against interview-led dietary recall.^20^ All patients were provided with examples, support, and guidance and following the LRD by a clinical dietician (PH). Further support for recording dietary intake or any other queries were supported by PH, DH, and AB, who were contactable throughout the study duration. Safety was based on the number of adverse events reported. All outcomes were compared between baseline and following 12 weeks of the LRD, except for the neurological bowel dysfunction (NBD) score and Newcastle Mitochondrial Disease Scale for Adults (NMDAS). The NMDAS is a qualitative rating scale that encompasses all aspects of mitochondrial disease, including GI symptoms and the quality of life that should be assessed every 6–12 months.^21^

### Gut Microbiome Profiling

DNA was extracted from 350 mg of stool collected from 10 patients at random and 10 control subjects, detailed in the Supplemental Appendix. Briefly, sequencing quality was assessed using FastQC, and quality scores were high (Phred > 28) across the length of all paired-end reads in all samples. Illumina adapters were trimmed using Trim Galore. Sequences were then filtered to remove rRNAs using SortMeRNA v2.1b to identify reads aligning to any of the included rRNA databases.^22^ Host-derived sequences were then filtered using Bowtie2 to remove reads aligning to the hg38 reference human genome.^23^ Filter summaries can be found in (Figures A1–A3). After filtering, samples, not including the negative control, had 11,935,479 ± 1,605,354 reads (mean ± standard deviation). A detailed description of methods for raw sequencing, profiling, and functional relative abundances is included in Supplemental Appendix (Figures A1–A3).

### Power and Statistics

Paired t-tests were used to compare within-group differences between before and after LRD intervention. To investigate changes between before and after LRD intervention, ordinal chi-square tests were performed on stool consistency (based on the ROME III cutoff values for stool consistency) and PAC-SYM data, and standard chi-square was used to compare changes in ROME III criteria. Pearson correlation coefficients were used to investigate associations between total NMDAS and NBD score, PAC-SYM, ROME III, stool frequency, and stool consistency. Sample size calculations for future studies were based upon the observed effect sizes (ESs) for the chi-square tests (Cramér's V) and calculated using the pwr library in R and detailed in the supplementary (page 6, lines 173–179). A full description of meta-genomic analysis, including data quality checks and correlations between study outcomes and gut microbiome, is presented in the Supplemental Appendix. All authors had access to the study data and reviewed and approved the final manuscript.

## Results

Between September 2017 and July 2018, 28 patients were enrolled into the study; 24 patients completed 12 weeks of LRD intervention. Patient characteristics are summarized in Table, with additional clinical data presented in Table A1. No significant differences in patients’ anthropometric, sleep duration, or physical activity were observed between before and after LRD intervention (Table).

**Table:** Data Are Mean (± SD) or n (%) Characteristics at Baseline and Following 12 wk of the Low-residue Diet for mtDNA-Related Disease Patients

Parameter	Pre (n = 24)	Post (n = 24)	*P* value
Anthropometrics			
Age (y)	52 (±14)		
Gender (female/male)	16/8		
Height (cm)	167 (±12)		
Weight (kg)	69 (±17)	70 (±19)	.10
BMI (kg/m^2^)	25 (±6)	26 (±6)	.13
Waist/hip ratio	0.96 (±0.09)	0.94 (±0.09)	.21
Physical activity/24 h (millig)	30 (±12)	28 (±8)	.73
Sleep duration/24 h	453 (±88)	432 (±93)	.51
Neurological bowel dysfunction:			
Very minor	6/24 (25%)		
Minor	4/24 (17%)		
Moderate	6/24 (25%)		
Severe	6/24 (25%)		
DNC	2/24 (8%)		
Blood biochemistry			
c-Peptide (nmol/mmol)	0.90 (±0.50)	0.90 (±0.40)	.50
Estimated glomerular filtration rate (ml/min/1.73 m^2^)			
<60	3 (13%)	2 (8%)	
>60–75	1 (4%)	1 (4%)	
>75	3 (12%)	21 (88%)	
Albumin (g/L)	49 (±8)	46 (±12)	.10
Alkaline phosphate (U/L)	69 (±18)	68 (±34)	.33
Alanine aminotransferase (U/L^−1^)	21 (±11)	23 (±9)	.47
Low-density lipoprotein (mmol/L)	3.4 (±1.10)	3.5 (±1.2)	.21
High-density lipoprotein (mmol/L)	1.4 (±0.30)	1.4 (±0.4)	.20
Triglycerides (mmol/L)	2.2 (±0.90)	2.1 (±0.8)	.20
Clinical features associated with mitochondrial disease			
Disease burden measured by the total NMDAS score	30 (±15)		
Swallowing	7/24 (29%)		
Gastrointestinal	18/24 (75%)		
Seizures	3/24 (13%)		
Stroke-like episodes	3/24 (13%)		
Diabetes	15/24 (63%)		
Questionnaires			
PAC-SYM	1.7 (±0.8)	1.1 (±0.9)	.03∗
Rome III criteria (≥3 criteria)	3.7 (±1.9)	2.7 (±1.6)	.01^#^
Bowel movement measures			
Stool frequency mean	3.5 (±1.4)	3.5 (±1.5)	1.00
Stool frequency total	92	80	.40
Total stool output over 5 d (grams)	294 (±204)	266 (±209)	.49
Colonic transit			
Normal <20%	3/24 (13%)	2/24 (8%)	
Delayed ≥20%	19/24 (79%)	20/24 (84%)	
DNC	2/24 (8%)	2/24 (8%)	
Colonic transit (ROM retained)	12.4 (±6.2)	11.3 (±7.4)	.40
Bristol stool consistency score (frequency)			
Hard/constipated 1–2	35	32	
Normal 3–5	33	39	
Loose stool 6–7	24	9	
Bristol stool score range	2.8 (±1.7)	2.1 (±1.5)	.08
Laxatives			
Osmotic total daily use			
Macrogol (Laxido^a^/Movicol^b^) (sachets)	31 (14)	18 (10)	
Lactulose (ml)	75 (2)	60 (2)	
Stimulant total daily use			
Senna (mg)	180 (2)	105 (1)	
Docusate (mg)	1200 (4)	1700 (7)	
Dulcolax Pico (ml)	55 (6)	10 (1)	
Sodium picosulfate (mg)	0 (0)	10 (1)	
Serotonin 5HT4-receptor agonist the total daily use			
Prucalopride (mg)	1 (1)	1 (1)	
Bicyclic fatty acid derived from prostaglandin E1 total daily use			
Lubiprostone (mcg)	24 (1)	24 (1)	

Laxatives presented as total daily usage (number of patients using laxatives). Bristol stool score grouping is based on ROME III criteria and is the frequency of each score. ∗ and ^#^ denote a significant difference at <0.01 and 0.05, respectively. ^a^ and ^b^ denote 13.125 and 13.8 g sachets. The threshold for individual clinical features to be interpreted as a binary trait, previously described.^3^

BMI, body mass index; cm, centimeters; DNC, did not complete; g, grams; kg, kilograms; mcg, millicentigram; mg, micrograms; ml, milliliter; mmol, millimoles; pg, pictograms.

### LRD Tolerability

Tolerability was assessed by food diaries and demonstrated a significant −34%-fold change in dietary fiber intake, reducing from 18 ± 8 g/d to 12 ± 6 g/d from baseline to study completion (mean reduction: 5 ± 10 grams per day, *P* = .03) (Table A3 and Supplemental Appendix). No changes were observed in any other dietary measures between before and after intervention (Table A3, *P* > .05). Four patients did not complete the study intervention, reporting the diet as too restrictive (n = 2) or due to health-related problems that were not GI-related (anxiety [n = 1] and muscle pain [n = 1]). No adverse events were reported in the remaining 24 patients who completed the 12-week LRD intervention.

### Bowel Movements

**Figure 2:**
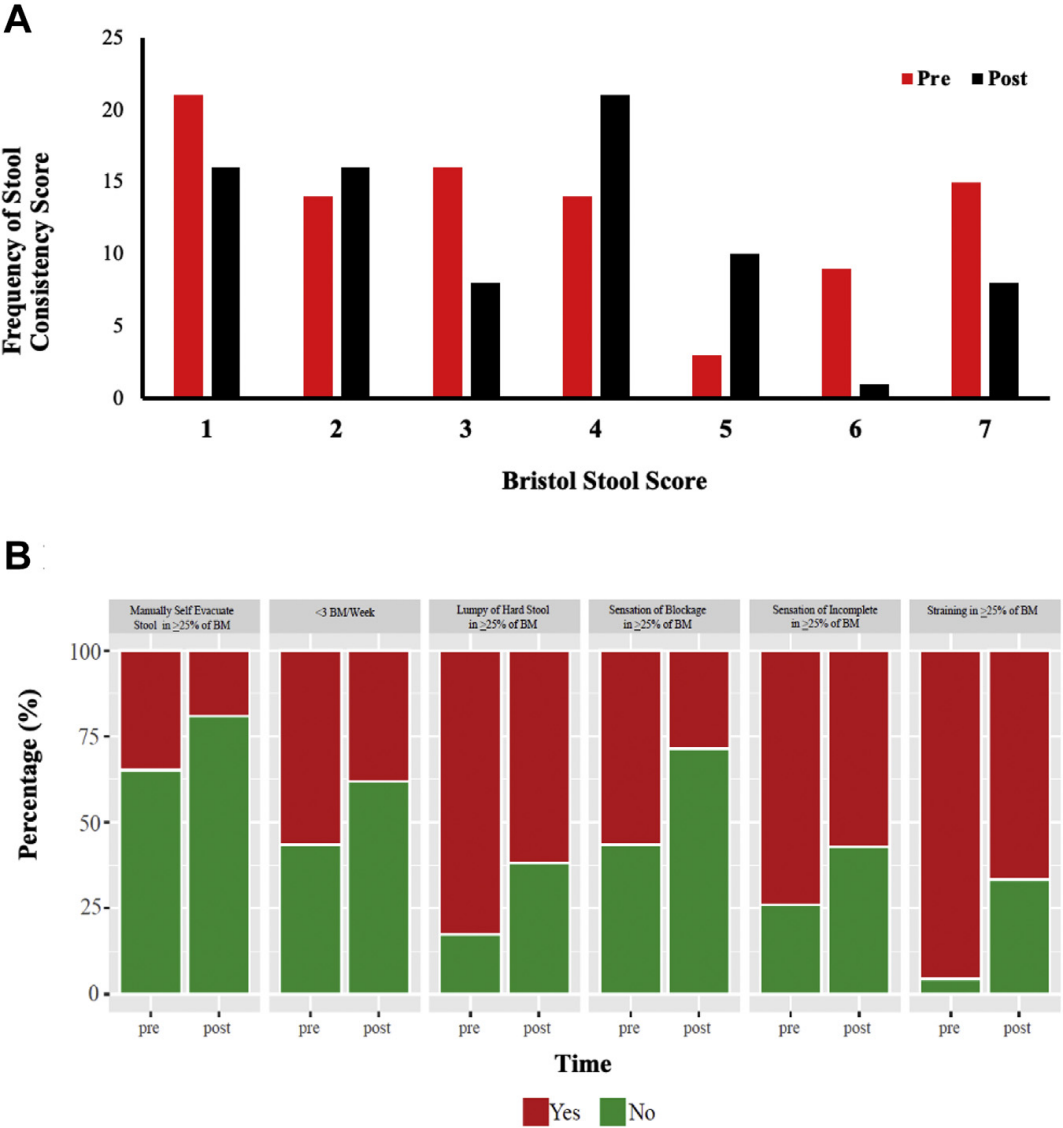
Pre- and post-LRD cumulative frequency of stool consistency based on (A) Bristol stool score ROME III (1–2: constipation, 3–5: normal stool, and 6–7: loose stools) and (B) proportional changes in ROME III criteria before and after LRD. BM, bowel movement (n = 24).

**Figure 3:**
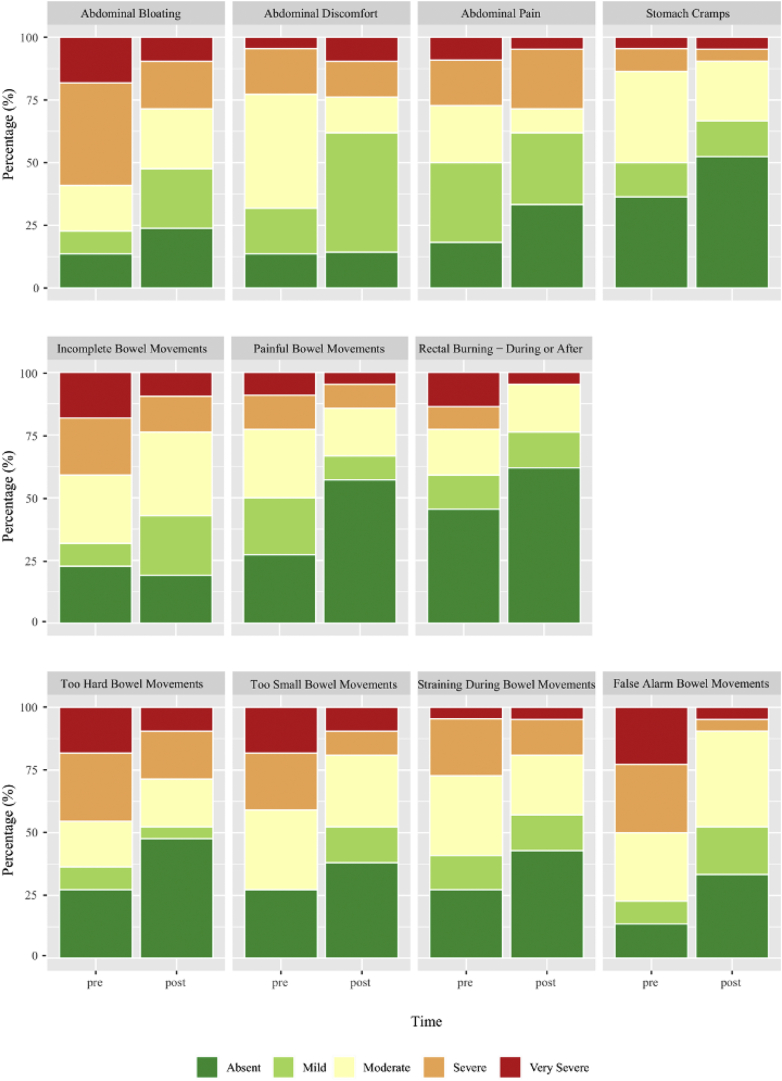
Pre- and post-proportional changes in Patient Assessment of Constipation-Symptoms (PAC-SYM). (*P* = .03), rectal (*P* = .03), and stool (*P* < .01) (n = 24).

NBD scores are detailed in Table, where the median NBD score for patients was 12 (range = 5–27). There was no significant change in the mean (*P* = 1.00) or total stool frequency (*P* = .40). There was a significant increase in the proportion of patients with normal stool and reductions in hard/constipated and loose stools when grouping stool consistency scores based on the ROME III cutoff values of 1–2 being constipation, 3–5 being normal, and 6–7 being loose stool/diarrhea (*P* = .01) (Table, Figure 2A) and a reduction in the range of stool consistency (−0.9 ± 1.3 [*P* = .08]) (Table, Table A2, Figure A1 and Supplemental Appendix). There were significant reductions in the mean PAC-SYM and ROME III scores of −0.5 ± 1.1 (*P* = .03), −1.0 ± 1.8 (*P* < .01) (Table). A significant reduction in the proportion of responses meeting ROME III criteria (*P* < .01) (Figure 2B) and in severity in all 3 subcategories of the PAC-SYM, abdominal (*P* = .03), rectal (*P* = .03), and stool (*P* < .01) (Figure 3) was observed. Based on the changes observed in PAC-SYM and ROME III, we calculated the sample sizes required to power future trials investigating the efficacy of an LRD (Figure A6). Using Cramér's V as the ES, we estimated the total sample size required to achieve 80% power with an alpha of 0.05 in a randomized controlled trial. Based on the changes observed in stool consistency, we would require 85 patients to detect an ES of 0.3; for the total PAC-SYM score, an ES of 0.2 would require 183 patients; for individual sections of the PAC-SYM data, 173 patients would be needed for the abdomen section (ES = 0.2), 210 patients for the rectal section (ES = 0.2), and 117 for the stool section (ES = 0.3). For the ROME III criteria data, 169 patients would be required (ES = 0.2). Four different laxatives were routinely used by patients (Table). Radiological evidence of delayed GI transit (≥20% of ROM retained in the GI tract) was observed in 19 patients at baseline and 20 patients at 12 weeks (See Figure A2 and Supplemental Appendix). No significant changes were observed in the CTT (*P* = .40) or total stool output (*P* = .49) following the LRD (Table). The number of patients and daily use of osmotic and stimulant laxatives were reduced following the LRD intervention (Table); however, no statistical tests were conducted due to the small sample size.

There was a direct relationship between the NBD score and the total NMDAS at baseline (*P* < .01, r = 0.61). An inverse association was observed between the worst score for stool consistency and ROME III (*P* = .02, r = −0.46) and PAC-SYM (*P* = .03, r = −0.45) at baseline and the worst score for stool consistency and ROME III at 12 weeks (*P* < .01, r = −0.71) (scores of 1 and 2 were deemed the worst and second worst score, respectively, based on patients' dysmotility). A direct relationship was observed between PAC-SYM and ROME III at baseline (*P* < .01, r = 0.72) and 12 weeks (*P* = .04, r = 0.42).

### Gut Metabolites and Blood Biochemistry

**Figure 4:**
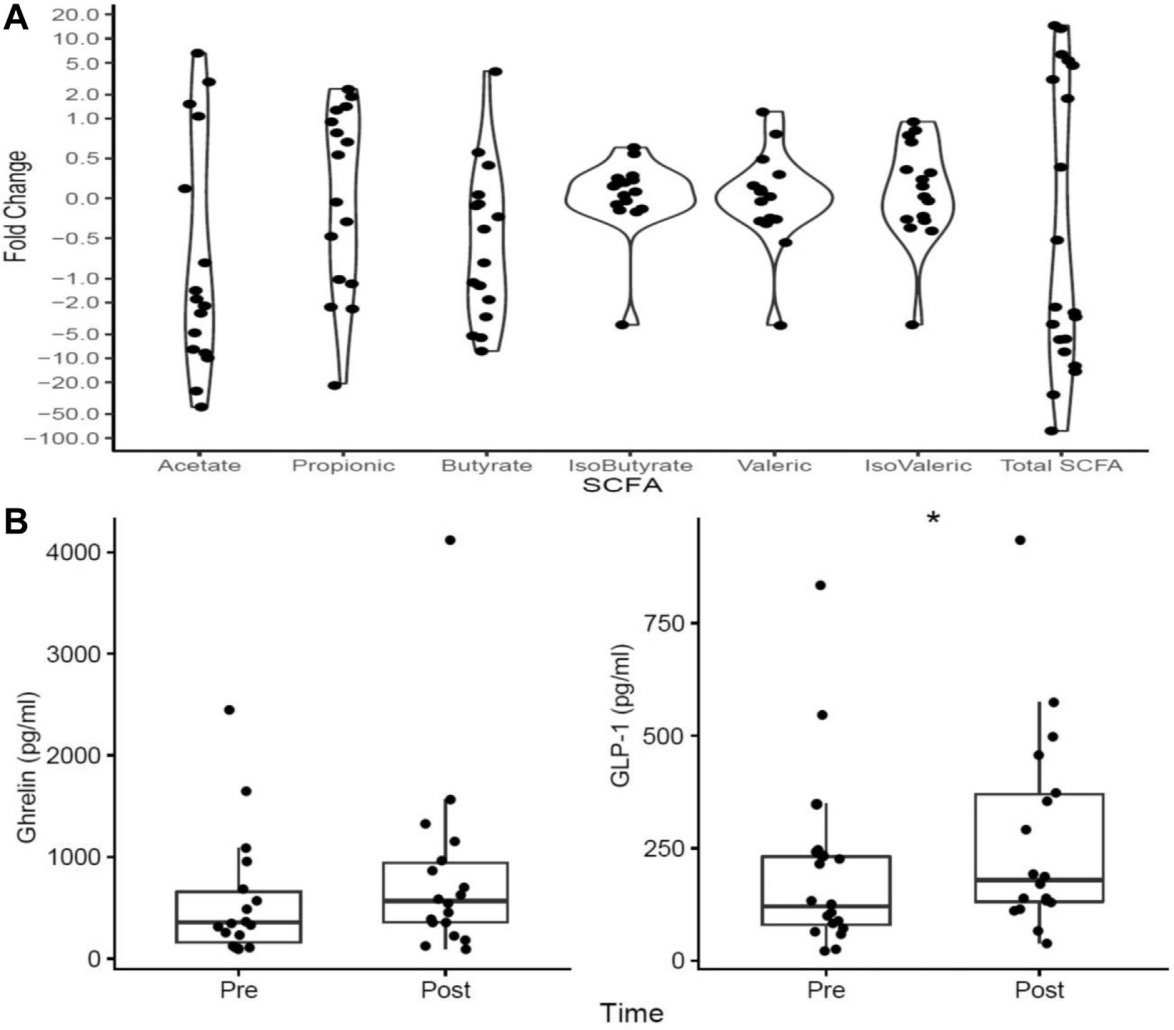
The violin dot plot of (A) changes in SCFA concentrations and (B) changes in ghrelin and glucagon-like peptide-1 (GLP-1) (n = 24).

No differences were observed in clinical blood biochemistry (Table). Among all the GI hormones and peptides assessed, only the glucagon-like peptide-1 level was significantly increased after LRD (*P* = .01) (Table A3, Supplemental Appendix and Figure 4B). The 3 main short-chain fatty acids (SCFAs), acetic acid (−3.7 ± 14.0 mmol/L) (*P* = .08), propionic acid (−0.5 ± 2.4 mmol/L) (*P* = .61), and butyric acid (−0.9 ± 1.7 mmol/L) (*P* = .06), and total SCFA concentrations (−5.2 ± 7.5 mmol/L) (*P* =.18) were all reduced in patients following the LRD, although these changes were not statistically significant (Table A3, Supplemental Appendix and Figure 4A).

### Gut Microbiome Diversity of Patients vs Control Subjects

In terms of species alpha diversity, no significant difference in the species estimates of Chao-1 (Mann-Whitney U, *P* = .05) or Shannon indices (Mann-Whitney U, *P* = .85) was observed between patients and controls subjects (Figure A8A). Similarly, no significant differences in UniFrac weighted (*P* = .19) or unweighted (*P* = .08) beta diversity (Figure A8B) were observed between patients and control subjects, and they were observed by permutational analysis of variance adjusting for age and body mass index (the mean age and body mass index were significantly different between patients and control subjects; 52 (± 14) vs 60 (± 10) years (*P* = .01) and 25 (± 6) vs 27 (± 3) kg/m^2^ (*P* = .05), respectively).

### Taxonomic Profiles of the Gut Microbiome

**Figure 5:**
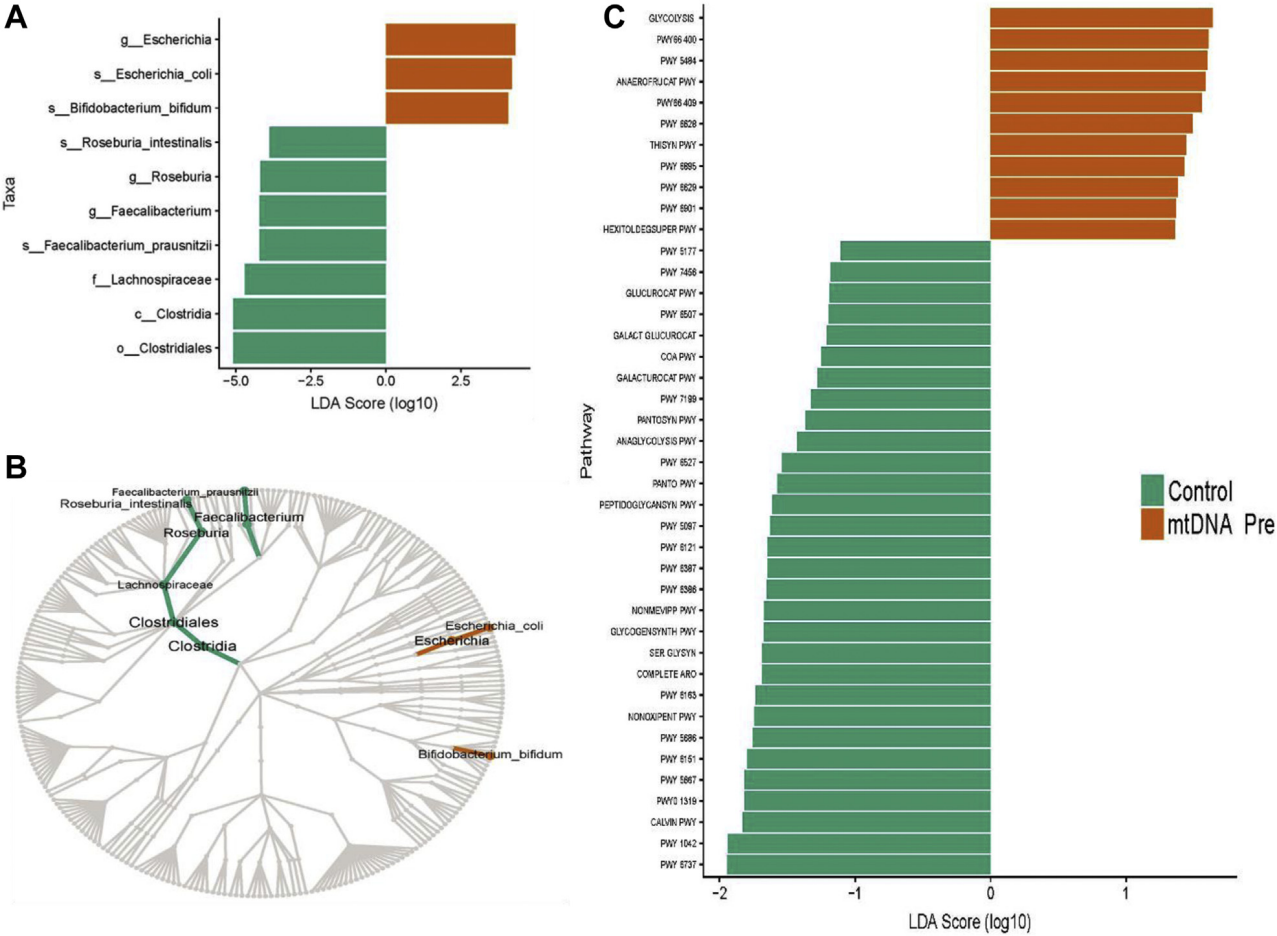
(A) Boxplots showing linear discriminant scores (LDAs) for significantly different bacterial abundances. (B) Cladogram of significantly different taxonomic differences. (C) Boxplots showing LDAs for significantly different functional profiles. The microbiome profile for controls (n = 10) and mtDNA-related disease patients (n = 10).

Taxonomic profiles of all samples reflected a composition expected for human gut microbiome samples (Figure A7 and Supplemental Appendix). No significant differences in taxa at any level were observed between male and female patients. Taxa at all levels of classification were tested for differential abundance between pre-LRD intervention patients and control subjects. At the species level, the mean relative abundance of *Escherichia coli* (3.7 ± 4.1 vs 0.6 ± 1.2) and *Bifidobacterium bifidum* (2.2 ± 1.9 vs 1.6 ± 1.7) was significantly higher (analysis of the composition of the microbiome [ANCOM] W > 0.6) in patients than that in control subjects (Figure 5A). Conversely, the abundances of *Faecalibacterium prausnitizi* (5.0 ± 3.4 vs 2.0 ± 3.1) and *Roseburia intestinalis* (2.0 ± 2.1 vs 0.5 ± 0.8), established butyrate-producing species, were significantly higher (ANCOM W > 0.6) in control subjects than those in patients (Figure 5A).

### Functional Profiles of the Gut Microbiome

Numerous functional pathways were identified that were significantly different (ANCOM W > 0.6) between patients and control subjects (Figure A7C) (full pathway names in Table A3). Among these, the greatest difference was a lower relative abundance of genes associated with starch degradation in patients than in control subjects. Patients additionally had a significantly lower relative abundance of several other metabolic pathways (Supplemental Appendix), suggestive of a divergent metabolic repertoire in patients’ gut microbiota. Indeed, the 3 significant pathways most enriched in patients were those associated with simple sugar metabolism (glycolysis pathways I, II, and IV), with this shift toward glycolysis pathways predominantly driven by the higher *E coli* abundance observed in Figure A9 and Supplemental Appendix.

### Gut Microbiome Changes Before and After LRD in Patients With mtDNA-Related Disease

The LRD intervention had no significant effect on alpha or beta diversity (Figure A11A and B and Supplemental Appendix Results). When considering taxonomic comparisons, 2 species, *Ruminococcus bromii* and *Alistipes putredinis*, had a significantly (ANCOM W > 0.6) higher relative abundance after LRD intervention (Figure A12 and Supplemental Appendix). No significant differences in the abundances of functional pathways were observed in the gut microbiome following the LRD intervention.

We explicitly compared species that were significantly different in the comparison between control subjects and patients in the patient stool before and after implementation of an LRD, to assess the impact of the intervention on these taxa, but observed no changes in their relative abundance after LRD intervention (Figure A10C and Supplemental Appendix). Additionally, we observed no significant difference in the beta diversity distances between the pre- and post-LRD intervention stool samples when compared to control subjects (Figure A12 and Supplemental Appendix). ROME III at baseline was positivity associated with *R intestinalis*, but no other associations were observed between bacterial abundances and bowel movements, GI symptoms, and ROME III criteria (Figure A14 and Supplemental Appendix).

## Discussion

Our findings demonstrate that an LRD is a safe and effective treatment for severe constipation symptoms such as abdominal distension, pain, bowel urgency, and diarrhea in a well-characterized cohort of patients with mtDNA-related disease. The LRD was well tolerated, as evidenced by reductions in dietary fiber and SCFA concentrations. Stool consistency improved and translated into a reduced use of laxatives, with good compliance, no reported adverse events, and no negative impact upon blood biochemistry or nutritional intake over the timespan of the intervention. These findings corroborate previous studies in other disease states, where low-residue (fiber) diets have shown promise in alleviating abdominal urgency, pain, distension,^16^ and constipation.^17^

GI dysmotility symptoms in mtDNA-related diseases are a common debilitating clinical manifestation in a disease where treatment strategies remain largely symptomatic.^24^ Alleviation of GI mechanical irritation and symptoms associated with GI dysmotility,^15^ in the absence of a change in the number of ROM in our study, provides insight into the potential mechanism of action of an LRD in mitochondrial disease. In our study, we observed reductions in dietary fiber and SCFA concentrations, indicative of fiber fermentation and gas production. In healthy subjects, gas production and retention have been shown to inhibit GI transit, decrease bolus propulsion, and elicit GI symptoms such as abdominal distension and pain.^25^ We propose that the short-term rescue of GI mechanical irritation and symptoms, bloating, abdominal pain, and stomach cramps associated with GI dysmotility is directly attributable to the reduced fiber intake, gas production, and potentially a reduction in the GI workload.^15^ Moreover, we suggest that our findings could better inform revision of expert opinion guidelines (eg, “https://www.newcastle-mitochondria.com/wp-content/cache/all/clinical-professional-home-page/clinical-publications/clinical-guidelines/index.html”), where an LRD may form part of a multidisciplinary approach to treat GI dysmotility and debilitating GI symptoms in this patient population and other disease states manifesting mitochondrial dysfunction.^7^

Although the LRD provides short-term rescue of GI symptoms, the pathological mechanisms responsible for GI dysmotility in mtDNA-related disease remain elusive, in part due to complex disease heterogeneity.^1^ Mitochondrial dysfunction in GI smooth muscle has been proposed,^5^ suggesting that enteric myopathy may underlie severe GI dysmotility, akin to cases of chronic intestinal pseudo-obstruction due to other etiologies.^2^^,^^5^ The use of CTT as an objective measure of GI motility identified significant GI dysmotility in mtDNA-related disease patients, and the retention of ROM may have important implications and guide clinicians in directing treatment approaches. However, the use of CTT is limited when differentiating between different forms of GI disorders and provides limited information relating to the pathophysiology of GI dysmotility.^26^ Moreover, it is important to acknowledge that GI symptoms, such as constipation, diarrhea, and bloating, are not always associated with gut dysmotility,^27^ suggesting that the GI symptoms experienced by patients in this study may not be specific to the lower GI tract or to GI dysmotility. A number of patients had persistent loose stool throughout the study. While this could simply relate to chronic laxative use, small intestinal bacterial overgrowth and deleterious changes in the gut microbiome, as observed here, should also be considered. Moving forward, alternative techniques to assess whole GI transit, potentially involving several modalities, inclusive of the upper and lower GI tract, to distinguish between dys-synergic defecation, colonic inertia, and proximal colon emptying may further our understanding regarding pathological mechanisms and guide clinical management.

Our study provides a novel insight into the gut microbiome of patients with mtDNA-related disease, implicating a higher relative abundance of *E coli* as responsible for modulating the metabolic capabilities of the microbiome. We observed a significant preferential switch from starch and complex carbohydrate degradation pathways to simple sugar pathways in patients with mtDNA-related disease. The gut microbiome profiles of patients observed here are unlikely to be transient, driven by the patient’s dietary intake and/or clinical phenotype^28^ and could be shaping the gut microbiome profile toward one that resembles inflammatory GI conditions.^29^ Similar increased *E coli* abundance has also been observed in Parkinson’s disease, where it was associated with increased gut permeability, serum markers of endotoxins, and increased alpha-synuclein.^30^ It is not clear whether such microbiome alterations are causative or a result of changes in GI dysmotility. However, preclinical models of primary mitochondrial disease hint at a pathological role, where *E coli* virulence determinants contributed to myenteric neuropathy^31^ and inhibition of neuronal activity associated with GI transit.^32^ These potential microbiome-related etiopathologies warrant further exploration in mtDNA-related disease.

Diet, a key feature that has been shown to rapidly and reproducibly modulate the gut microbiome,^33^ could also contribute to microbiome-related etiopathologies. Although modest, the LRD was able to moderately increase the relative abundance of *R bromii* and *A putredinis* in patients, 2 species that have been shown to be involved in carbohydrate metabolism.^34^^,^^35^ The increase in *R bromii* and *A putredinis* is likely to be due to baseline bacterial composition and/or due to the ability of *R bromii* and *A putredinis* to outcompete other strains of bacteria for what little dietary fiber remained in patients' diets, colonizing and therefore increasing their relative abundance.^35^ Our study provides novel findings that stimulate the need for a more focussed approach for the clinical management of GI dysmotility in mtDNA-related disease patients, potentially utilizing dietary interventions that can increase the abundance of bacteria involved in SCFA production, such as *F prausnitzii* and *R intestinalis*, while outcompeting species such as *E coli* to improve the patient’s GI dysmotility and clinical outcomes.

Early satiety, commonly reported in mitochondrial disease,^1^ may also impact upon GI motility. Low concentrations of SCFAs may impair neurotransmitter release in the GI tract, such as serotonin,^36^ exacerbating GI dysmotility through increased glucagon-like peptide-1 levels, evident in preclinical models, corroborating our findings.^37^ Second, prolonged low intake of fiber may impair the GI tract's ability to process fiber, further reducing the relative abundance of bacteria involved in fermentation (*F prausnitzii* and *R intestinalis*). This, in turn, may add bulk to the digesta, potentially increasing GI workload, mechanical irritation, and GI symptoms.^15^ Third, chronic fiber deficiency may impair GI integrity, where the gut microbiome switches its preferred energy source to glycoproteins secreted by the mucus barrier, evident in germ-free mice.^38^ This may be exacerbated further due to low concentrations of SCFAs, important for their anti-inflammatory properties and ability to modulate potentially pathogenic bacteria such as *E coli*,^39^ which has been linked with the disease onset and progression in IBS.^40^ Our data provide further evidence that the pursuit of microbiome-targeted therapeutics, although challenging, may benefit patients with neurological disorders manifesting with debilitating GI dysmotility by providing the potential beneficial effects of fiber while maintaining the improvements in GI symptoms observed here.

### Limitations

There are several limitations in this study. First, the small sample size and multiple outcome measures explored were due to the largely unknown ESs of the chosen outcomes in mtDNA-related disease. Second, no control arm was included in the intervention part of the study, meaning that a placebo effect cannot be excluded. However, the use of objective measures (CTT, gut microbiome, and metabolites) reduced subjective interpretation and recall bias. Further, while we observed a positive association between NBD and NMDAS scores, suggesting that the severity of GI dysmotility was proportional to the overall disease burden, the exploratory nature of this study precluded robust assessment of NBD and/or NMDAS as predictors of favorable response to the LRD intervention. Due to the exploratory nature of this study, NBD was only performed prior to the LRD; therefore, if the relationship between overall disease burden of mtDNA and NBD was present following the LRD, it cannot be concluded. Assessing NBD before and after any intervention will be included moving forward. Careful selection for suitable control subjects was considered to mitigate environmental factors; however, a more suitable control group such as Parkinson disease patients may provide greater insight into pathological mechanisms responsible for GI dysmotility and associated symptoms. Finally, the small change we observed in the gut microbiome in patients following the LRD may be in part due to the already low levels of dietary fiber being consumed at baseline, which are lower than the UK-based recommendations of >30 g/d. However, the question remains if a −34%-fold decrease in dietary fiber observed here would have affected the gut microbiome in other pathologies, or if the lack of change in the gut microbiome following the LRD is just a reflection of the complex phenotype and lifestyle of mtDNA patients. Although we observed significant differences in the gut microbiome between mtDNA patients and controls, these are relative abundances, and moving forward, quantitative polymerase chain reaction would be useful to assess taxonomic abundances.

## Conclusion

In summary, our findings show significant promise for the use of an LRD to improve GI symptoms in patients with mtDNA-related disease and chronic constipation. Early intervention with an LRD for bowel urgency, diarrhea, pain, and distension and strategies to mitigate their progression might improve GI outcomes in patients with mtDNA-related disease.

## Supplementary Materials

Supplementary Material

CONSORT-2010-Checklist
