# Supplementary material for: Phase II Feasibility Study of the Efficacy, Tolerability, and Impact on the Gut Microbiome of a Low-Residue (Fiber) Diet in Adult Patients With Mitochondrial Disease
Source: Gastro Hep Adv. 2022 Jul 1;1(4):666–77. doi: 10.1016/j.gastha.2022.03.007 (PMC11307892; doi:10.1016/j.gastha.2022.03.007)
Supplement: Supplementary Material [file mmc1.docx]

**Supplemental Appendix**

Phase II feasibility study of the efficacy, tolerability, and impact on the gut microbiome of a low residue (fibre) diet in adult patients with mitochondrial disease.

**Contents**

[**Methods** 3](#_Toc59104943)

[**Participants** 3](#_Toc59104944)

[**Procedures** 3](#_Toc59104945)

[**Outcomes** 3](#_Toc59104946)

[**Stool Metabolites** 4](#_Toc59104947)

[**Gastrointestinal Hormones and Peptides** 4](#_Toc59104948)

[**Statistics** 4](#_Toc59104949)

[**Gut Microbiome Profiling** 4](#_Toc59104950)

[**Metagenomic diversity analyses** 5](#_Toc59104951)

[**Metagenomic differential relative abundance analyses** 5](#_Toc59104952)

[**Results** 6](#_Toc59104953)

[**Supplemental Tables** 7](#_Toc59104954)

[**Table S1**: Demographic summary of mtDNA-related disease patients 7](#_Toc59104955)

[**Table S2**: Stool Characteristics During Five Days of Whole Gut Transit Assessment 9](#_Toc59104956)

[**Table S3:** Data are mean (± SD) characteristics at baseline and following 12-weeks of the Low Residue Diet for mtDNA-related disease patients. 11](#_Toc59104957)

[**Table S4:** Functional pathway acronyms and full names. 12](#_Toc59104958)

[**Supplemental Figure Legends** 13](#_Toc59104959)

[**Figure S1.** 14](#_Toc59104960)

[**Figure S2.** 15](#_Toc59104961)

[**Figure S3.** 16](#_Toc59104962)

[**Figure S4.** 17](#_Toc59104963)

[**Figure S7.** 20](#_Toc59104964)

[**Figure S8.** 21](#_Toc59104965)

[**Figure S9.** 22](#_Toc59104966)

[**Figure S10.** 23](#_Toc59104967)

[**Figure S12**. 25](#_Toc59104968)

[**Figure S13.** 26](#_Toc59104969)

[**Figure S14.** 27](#_Toc59104970)

[**References** 28](#_Toc59104971)

**Methods**

**Participants**

Patients were eligible for inclusion if they met the inclusion detailed in the main manuscript and must be on a stable gastrointestinal (GI) drug regimen, (including no antibiotic use) for at least 3 months prior to commencement of the study; not pregnant or breastfeeding; competent to make decisions relating to the study and were able to provide informed written consent. Patients were excluded if they had a confirmed bowel obstruction; planned surgery and had begun a new drug regime or were participating in any drug study 3 months prior to study commencement and any existing conditions that may be exacerbated by taking part in the study. Eligible patients were identified and recruited between September 2017 and April 2018. Control subjects with no pre-existing diagnosis of mitochondrial or GI disorders and not already implementing a LRD were invited to participate in gut microbiome profiling at baseline only. Control subjects provided a stool sample only for gut microbiome profiling (at baseline only) and were partners or caregivers of patients enrolled onto the study.

**Procedures**

Patients provided a fasted blood sample to investigate GI hormones and peptides (detailed in the Supplemental Appendix); completed GI symptom questionnaires and were administered with an oral colonic marker capsule containing 20 radiopaque markers (ROM) during visit one to assess colonic transit. ^1^ Patients collected all stool passed using fecotainers; record total number of bowel movements using questionnaires; food frequency diaries, kept a sleep diary and wear a physical activity monitor between visits one and two.

Five days following visit one patients and control subjects returned for visit two and provided a 2-3g stool sample, which was then stored at -80˚C until further analysis. Patients also collected the total number of bowel movements, returned total stool passed; questionnaires; sleep diary and the physical activity monitor. Patients then attended the X-ray department to assess colonic transit, and where appropriate were instructed to abstain from the use of laxatives for two days prior to and during the assessment of transit time. Delayed colonic transit time (CTT) was defined as the retention of more than four ROM (≥20%) in the GI tract on day 5 after ingestion. ^2^ Each diet was personalised by a clinical dietician (PH) to the patient's current dietary intake to optimise nutritional intake in conjunction with taking a prescribed prescription of Forceval^®^, a multivitamin and mineral supplement), to take once a day. All primary and secondary outcomes measures were assessed at baseline and following 12 weeks of the LRD intervention, except the Newcastle Mitochondrial Disease Scale for Adults (NMDAS) and the Neurological Bowel Dysfunction (NBD) score (baseline only)

Following the 12-week LRD intervention, patients were invited back for visits three and four, which were the same as one and two, respectively (Figure 1). Intention to treat included all patients who completed the LRD, and all data collected prior to patients dropping out of the study (n=4), as laid out in the consent form.

**Outcomes**

Bristol Stool Score (BSS) were in accordance with the ROME III cut off values, ^3, 4^ clinical GI symptoms, using the ‘Patient Assessment of Constipation-Symptoms questionnaire’ (PAC-SYM), ^5^ ROME III criteria and CTT (determined by remaining ROM in GI tract) and total stool output passed over five days; GI metabolites (Short Chain Fatty Acids, (SCFA)), hormones (Ghrelin and Leptin) and peptides (Glucagon-like peptide-1 (GLP-1), Glucagon-like peptide-2 (GLP-2), Peptide YY (PYY), Vasoactive Intestinal Peptide (VIP) and Nestafin-1); physical activity and sleep duration were recorded over seven to ten days using GeneActiv^®^ accelerometer, ^6^ dietary intake over 72 hours using food frequency diaries and uploaded into INTAKE24 ^7^ and microbiome profiles were all assessed at baseline and 12-weeks following a LRD (Figure 1). Controls provided a stool sample for gut microbiome analysis at baseline only. The gut microbiome profile of ten patients selected at random was compared with ten control subjects at baseline only.

**Gut Metabolites**

Analysis of short-chain fatty acids (SCFA) was performed using 250mg of stool as previously described. ^8^ 250mg of stool was added to a deproteinising solution at half weight by volume and mixed. 200µl of deionised water was added before being mixed and then centrifuged at 15,000rpm for 20 minutes. The supernatant was removed and then analysed using gas chromatography (Shimadzu GC-2014 with AutoInjector AOC-20i, Japan). 5g of stool collected for each patient at baseline and following 12 weeks of the LRD was aliquoted and stored at -80˚C, and the remaining was disposed of.

**Gastrointestinal Hormones and Peptides**

Analysis of GI hormones and peptides were all assessed using ELISA, as per the manufacturer’s instructions; Ghrelin (Merck Millipore, EZGRA-88K), Leptin (Thermo Fisher Scientific, KAC2281), Glucagon-like peptide-1 (GLP-1) (Thermo Fisher Scientific, EHGLP), Glucagon-like peptide-2 (GLP-2) (LSBio, LS-F6546), Vasoactive Intestinal Peptide (VIP) (LSBio, LS-F4057), Nestafin-1 (Abcam, ab213812) and Peptide YY (PYY) (Merck Millipore, EZHPYYT66K). All analysis and processing of blood samples was performed by the same member of the research team who was blind to the time point of the sample being analysed.

**Statistics**

Pearson correlation coefficient was used to investigate associations pre and post LRD between total NMDAS and NBD score, PAC-SYM, ROME III, stool frequency and stool consistency (worst score, where 1 and 2 were the worst, followed by 7 and 6, and finally 3, 4 or 5). Sample size calculations for future studies were based upon the observed effect sizes for the chi-square tests (Cramér's V) and calculated using the pwr library in R. A full description of metagenomic diversity, differential relative abundance analyses, including data quality checks (Figure S3, S4 and S5, Supplemental Appendix) and spearman correlations between BSS, PAC-SYM, NBD, ROME III and the gut microbiome can be found in the Supplementary Appendix.

**Gut Microbiome Profiling**

DNA was extracted from 350mg of stool sample using the Powerlyzer Powersoil DNA isolation kit (MoBio, UK), following manufacturer’s instructions and as previously described. ^9^ DNA quantity was confirmed using the Qubit dsDNA broad range Assay Kit (Invitrogen) and the NanoDrop™ One Spectrophotometer (Thermo scientific) while DNA integrity was assessed visually by gel electrophoresis. Multiplexed metagenomic sequencing libraries were prepared by 251-bp paired-end protocols and sequenced on a HiSeq. 2500 (Illumina Inc.) platform at the Singapore Centre for Environmental Life Sciences Engineering, Nanyang Technological University (Singapore).

Taxonomic profiling of samples was carried out using MetaPhlAn2 to align reads from both paired-end files to taxonomically unique marker genes, ^10^ returning relative abundances at all taxonomic levels from phylum to species. HUMAanN2 was used (again using both paired-end read files) to estimate functional relative abundances using the ChocoPhlAn and full UniRef90 databases, returning MetaCyc pathway level relative abundances per taxa. ^11^

**Metagenomic diversity analyses**

Within sample taxonomic diversity (alpha diversity) of each sample was estimated using the Chao1 and Shannon indices. These were generated using the *‘estimate_richness’* command from the Phyloseq package in R from pseudo counts generated by multiplying the estimated species relative abundances produced by MetaPhlAn2 by 100,000. ^12, 13^ Significant differences in alpha diversity measures, age, and BMI between controls and patients and were assessed using Mann-Whitney-Wilcoxon tests in R with a threshold of *p*<0·05. Both weighted and unweighted UniFrac beta-diversity measures were calculated from pseudo counts using the Phyloseq *‘UniFrac’* command and the MetaPhlAn2 phylogenetic tree within the curated MetagenomicData package. ^14^ Ordination coordinates for both measures were generated using the Phyloseq *‘ordinate’* command to carry out multidimensional scaling. Permutational analysis of variance (PERMANOVA) was used to model the association of age, BMI and diseases status (control subject or patient) with both UniFrac distance measures (variables provided in that order). This was carried out using the *’adonis’* command in the Vegan package using 100,000 permutations. ^15^ All comparisons to controls considered the pre-intervention samples for patients. In comparisons of pre and post-intervention samples, paired Wilcoxon signed-rank tests were used to assess differences in alpha diversity measures and patient was included as an additional covariate in the beta-diversity PERMANOVA analyses. Both weighted and unweighted UniFrac distances were also used to compare the similarity of pre and post-intervention samples to controls.

**Associations between Gut Microbiome and Questionnaire GI Symptoms and Bowel Movements**

Spearman correlations were calculated pairwise between the questionnaire derived scores (NMDAS total and GI score, Neurological Bowel Dysfunction Score, PAC-SYM, ROME III, Stool frequency and consistency) and both the relative abundance of significant species, as a percentage of the sample total, and measures of alpha diversity. This was carried out independently for the pre and post intervention patient microbiome samples. P-values were corrected for multiple testing using the Benjamini-Hochberg FDR adjustment, considering a significant correlation where p<0.1. Both questionnaire and microbiome features were ordered for visualisation using complete hierarchical clustering of Euclidean distances estimated from correlation estimates.

**Metagenomic differential relative abundance analyses**

Analysis of Composition of Microbiomes (ANCOM) was used to identify taxa significantly different between controls and mtDNA disease patients pre-intervention. ^16^ This looks for differences in the ratio of each taxa relative to all other taxa between the two groups and is robust to the compositional nature of relative abundance data. The latest R code (ANCOM 2·0) was obtained from the developer's website (<https://sites.google.com/site/siddharthamandal1985/research>). ANCOM was run including age and BMI as covariates and a false-discovery rate corrected *p*-value threshold <0·1 for considering individual ratio tests significant. A taxon was considered differentially abundant between the two groups if its ratio to at least 60% of other taxa considered was significantly different between them (ANCOM W>0.6). This is the most permissive of the three significance cut-offs provided within ANCOM and was selected given the small sample size (n=10) of each group. ANCOM was run at each taxonomic level independently, only considering taxa observed in at least 25% of samples. As ANCOM provides no estimate of relative effect sizes between significant associations, LEfSe was used to determine the direction and linear discriminant analysis score between groups for significant taxa identified using ANCOM. ^17^ Cladograms were plotted using the Metacoder package. ^18^ Differences in metagenomic functional potential between controls and patients were similarly assessed by similarly applying ANCOM and LEfSe to MetaCyc pathway-level relative abundances (not stratified by taxonomy). Taxonomic and functional differences between pre and post-intervention samples were also assessed using ANCOM and LEfSe in tandem but using the patient as a covariate in place of age and BMI in the ANCOM model. Plots summarizing the taxonomic contribution of each taxa to functional pathways in all samples were generated using the HUMAanN2 *‘humann2_barplot’* function. This trial is registered at ClinicalTrials.gov, number NCT03388528.

**Results**

No significant differences in patients’ anthropometric measurements including, body mass index (BMI) (p=0.13), waist to hip ratio (p=0.21), physical activity (p=0.72) or sleep duration (p=0.51) were observed between pre and post LRD intervention (Table 1).

**Gut Microbiome Profile of Patient vs. Control Subjects**

**Taxonomic Profiles of the Gut Microbiome**

Taxonomic profiles of all samples reflected a composition expected for human gut microbiome samples (Figure S7, Supplemental Appendix). Taxa at all levels of classification were tested for differential abundance between pre-LRD intervention patients and control subjects. At species level, the mean relative abundance of *Escherichia coli* (*E. coli*) (3·7 ± 4·1 vs. 0·6 ± 1·2) and *Bifidobacterium bifidum* (*B. bifidum*) (2·2 ± 1·9 vs. 1·6 ± 1·7) were significantly higher (ANCOM W>0.6) in patients when compared with controls (Figure 7A). Conversely, the abundances of *Faecalibacterium prausnitizi* (*F. prausnitizi*) (5·0 ± 3·4 vs. 2·0 ± 3·1) and *Roseburia intestinalis* (*R. intestinalis*) (2·0 ± 2·1 vs. 0·5 ± 0·8), well-known butyrate-producing species, were significantly higher (ANCOM W>0.6) in control subjects when compared with patients (Figure 7A).

**Functional Profiles of the Gut Microbiome**

The abundance of different functional pathways in the gut microbiome was also similarly compared between control subjects and patients. Numerous functional pathways were identified that were significantly different (ANCOM W>0.6) between patients and controls (Figure S7C) (full pathway names in Table S3. Patients additionally had a significantly lower relative abundance of several other metabolic pathways, including the pentose phosphate cycle, four amino acid pathways, three nucleotide biosynthesis pathways and two carbohydrate degradation pathways amongst others. Suggestive of a less diverse metabolic repertoire in patient gut microbiome. This shift in glycolysis pathways is almost all accounted for by the higher in *E.coli* relative abundance in patients, with the three pathways with the largest relative abundance illustrated in (Figure S8, Supplemental Appendix). Similarly, two amino acid pathways (tryptophan and phenylalanine biosynthesis) had a significantly higher relative abundance in patients (Figure S10, Supplemental Appendix).

**Gut Microbiome Changes Pre and Post LRD in patients with mtDNA-related disease**

**Taxonomic, Metabolic and Functional Profile of the Gut Microbiome**

There were no significant differences in the abundance of different functional pathways in the gut microbiome following a LRD (p>0·05). These results suggest that the LRD intervention influenced the taxonomic composition of the patients but only moderately, and did not reflect the differences in taxonomic composition observed between patients and controls pre-intervention. To confirm this, we compared the bacteria that were significantly different between controls and patients prior to the LRD and in the post LRD samples, to assess the impact of the LRD on these bacterial species. There was no significant changes in any of these bacterial species following a LRD, further indicating that the intervention does not make the patient gut microbiome more like that of the control subjects (p>0·05) (Figure 5C). Indeed, comparing the beta diversity distances between the pre and post LRD intervention samples to the control subjects, we observed no significant difference between controls and patients (p>0·05) (Figure S11, Supplemental Appendix). The LRD intervention had no significant effect on alpha (Mann-Whitney U Chao-1, p=1·00 or Shannon indices, p=0·41) or beta diversity (PERMANOVA Weighted, p=0·98 or Unweighted, p=0·34) between baseline and following 12-weeks of the LRD intervention (Figure S11A and S11B, Supplemental Appendix).

We additionally examined the bacteria that were significantly different between controls and patients before the LRD in the post LRD samples, to assess the impact of the LRD on these bacterial species but observed no changes in their abundance with the intervention (Figure S10C, Supplemental Appendix). Additionally, we observed no significant difference in the beta diversity distances between the pre and post LRD intervention samples when compared to the control subjects (Figure S13, Supplemental Appendix), indicating that the intervention is not shifting the patient microbiome to that of control subjects. ROME III at baseline was positivity associated with *R.intestinalis*, but no other associations were observed (Figure S14, Supplemental Appendix).

**Supplemental Tables**

**Table S1**: Demographic summary of mtDNA-related disease patients

| **Patient (Gender)** | **Age** | **Clinical Features** | **Genetic Defect** | **Muscle**  **Heteroplasmy (%)** | | **Blood**  **Heteroplasmy (%)** | | **Urine Heteroplasmy**  **(%)** |
| --- | --- | --- | --- | --- | --- | --- | --- | --- |
| 1 (M) | 49 | Deafness; Diabetes; Fatigue; Bowel/GUT dysmotility; Myalgia; Ptosis. | m.3243A>G | ·· | 31 | | 88 | |
| 2 (M) | 48 | Diabetes; Bowel/GUT dysmotility; Ataxia; Deafness; Migraine; Fatigue; Ptosis; CPEO. | m.3243A>G | 66 | 18 | | 80 | |
| 3 (F) | 61 | Deafness; Fatigue; Bowel/GUT dysmotility; Myalgia; Weakness/Myopathy; Dysphagia; Migraine; Ataxia; Extrapyramidal involvement. | m.3243A>G | 55 | 6 | | 40 | |
| 4 (F) | 56 | Deafness; Diabetes; Bowel/GUT dysmotility; Weakness/Myopathy; Ataxia; Neuropathy. | m.3243A>G | 67 | 15 | | 58 | |
| 5 (F) | 56 | Bowel/GUT dysmotility; Weakness/Myopathy; Fatigue; Dysphagia; Migraine; Ataxia. | m.1624C>T | ·· | ·· | | ·· | |
| 6 (F) | 61 | Deafness; Diabetes; Bowel/GUT dysmotility; Fatigue; Migraine; Ptosis; Neuropathy; Ataxia; Weakness/Myopathy. | m.3243A>G | 11 | 3 | | 14 | |
| 7 (F) | 67 | Deafness, Ataxia, Bowel/GUT dysmotility; Weakness/Myopathy; Diabetes; Dysphagia. | m.14709T>C | 87 | 75 | | 60 | |
| 8 (F) | 26 | Deafness; Diabetes; Weakness/Myopathy; Migraine; Dysphagia; Migraine; Ataxia; Bowel/GUT dysmotility. | m.3243A>G | 80 | 44 | | 65 | |
| 10 (F) | 57 | Deafness; Diabetes; Bowel/GUT dysmotility; Fatigue. | m.3243A>G | 73 | 6 | | 46 | |
| 11 (F) | 35 | Deafness; Diabetes; Bowel/GUT dysmotility. | m.3243A>G | ·· | 31 | | 55 | |
| 12 (F) | 72 | Fatigue, Ptosis, Dysphagia; Migraine; Seizures; Encephalopathic episodes; CPEO; Weakness/Myopathy; Bowel/GUT dysmotility. | m.12320A>G | N/A | ·· | | ·· | |
| 14 (F) | 60 | Ataxia; Migraine; Ptosis; Bowel/GUT Dysmotility. | m.3243A>G | ·· | 5 | | 25 | |
| 15 (M) | 67 | Ataxia; Diabetes; Bowel/GUT dysmotility; Deafness; Fatigue; Neuropathy; Weakness/Myopathy; Dysphagia; Dysphonia/Dysarthria. | m.3243A>G | 67 | ·· | | 66 | |
| 18 (M) | 68 | Ataxia; CPEO; Seizure; Diabetes; Cognitive impairment/dementia; Stroke-like episode; Bowel/GUT dysmotility; Ptosis; Deafness; Weakness/Myopathy; Cardiomyopathy; Dysphonia/Dysarthria. | m.3243A>G | 26 | 28 | | 87 | |
| 22 (F) | 47 | Ataxia; Deafness; Fatigue; Diabetes; Bowel/GUT dysmotility; Fatigue; Weakness/Myopathy; Neuropathy. | m.3243A>G | 77 | 17 | | 51 | |
| 23 (M) | 61 | Ataxia; Bowel/GUT dysmotility; Deafness; Fatigue; Weakness/Myopathy; Migraine; CPEO; Neuropathy. | m.13513G>A | 60 | ·· | | 42 | |
| 24 (M) | 42 | Ataxia; Cognitive impairment/dementia; Stroke-like episode; Dysphagia; Seizures; encephalopathic episodes; Bowel/GUT dysmotility | m.3243A>G | 92 | 41 | | 91 | |
| 26 (M) | 49 | Deafness; diabetes; Bowel/GUT dysmotility; Migraine | m.3243A>G | ·· | 28 | | 53 | |
| 27 (M) | 66 | Deafness; diabetes; Bowel/GUT dysmotility; cardiomyopathy; CPEO; Ptosis; Weakness/ Myopathy; Ataxia, Neuropathy; Dysphagia | m.3243A>G | ·· | 14 | | 64 | |
| 28 (M) | 54 | Deafness; diabetes; Bowel/GUT dysmotility; migraine; Ptosis; Weakness/ Myopathy; Ataxia, Neuropathy; Dysphagia; encephalopathic episodes; pyramidal involvement | m.3243A>G | 74 | 17 | | 76 | |
| 29 (F) | 32 | Deafness; Migraine; seizures; stroke-like episodes; encephalopathic episodes; Bowel/GUT dysmotility; Ptosis; CPEO; Dysphonia/Dysarthria; Weakness/Myopathy; Ataxia; Neuropathy; Pyramidal involvement | m.3243A>G | 50 | 21 | | 42 | |
| 30 (F) | 50 | Deafness; diabetes; Bowel/GUT dysmotility; Weakness/ Myopathy; Migraine; Dysphagia; Ptosis; Ataxia; Neuropathy | m.3243A>G | 75 | 19 | | 78 | |
| 32 (M) | 31 | Deafness; Migraine; Seizures; Bowel/GUT Dysmotility; Ptosis; Weakness/Myopathy | m.3243A>G | 89 | 31 | | 87 | |
| 33 (F) | 25 | Deafness; Bowel/GUT dysmotility; Ataxia | m.3243A>G | ·· | 36 | | 80 | |

M, male; F, female; N/A, not applicable. * N/A denotes a confirmed diagnosis of mitochondrial related disease but no level of heteroplasmy.

**Table S2**: Stool Characteristics During Five Days of Whole Gut Transit Assessment

|  | 5 Days During Whole Gut Transit Assessment | | | | | | | | | |
| --- | --- | --- | --- | --- | --- | --- | --- | --- | --- | --- |
|  | **1** | | **2** | | **3** | | **4** | | **5** | |
| Patient (gender) | **Weight (g)** | **BSS** | **Weight (g)** | **BSS** | **Weight (g)** | **BSS** | **Weight (g)** | **BSS** | **Weight (g)** | **BSS** |
| PRE |  |  |  |  |  |  |  |  |  |  |
| 1 (M) | 118 | 1/2* | 47 | 1/2* | 67 | 1/2* | 53 | 1 | ·· | ·· |
| 2 (M) | 109 | 3 | 109 | 3 | 142 | 3 | ·· | ·· | ·· | ·· |
| 3 (F) | 27 | 1 | 5 | 1 | 61 | 4 | 26 | 4 | 14 | 1 |
| 4 (F) | 102 | 5 | 27 | 6 | 4 | 6 | ·· | ·· | ·· | ·· |
| 5 (F) | 16 | 1/7* | ·· | ·· | ·· | ·· | ·· | ·· | ·· | ·· |
| 6 (F) | 204 | 1 | ·· | ·· | ·· | ·· | ·· | ·· | ·· | ·· |
| 7 (F) | 75 | 3 | 52 | 1 | 71 | 4 | 65 | 5 | 118 | 7 |
| 8 (F) | 32 | 6 | 11 | 1/2* | 31 | 3 | ·· | ·· | ·· | ·· |
| 10 (F) | 113 | 1/2/3/4* | 204 | 5/6/7* | ·· | ·· | ·· | ·· | ·· | ·· |
| 11 (F) | 78 | 1/2* | 90 | 3 | ·· | ·· | ·· | ·· | ·· | ·· |
| 12 (F) | 2 | 1/2* | 161 | 3 |  |  | 125 | 6 | 144 | 7 |
| 14 (F) | 68 | 1 | 19 | 1 | 150 | 1 | ·· | ·· | ·· | ·· |
| 15 (M) | 98 | 3 | 179 | 3 | 100 | 3 | ·· | ·· | ·· | ·· |
| 18 (M) | 127 | 4 | 1 | 4 | 91 | 4 | 167 | 4 | ·· | ·· |
| 22 (F) | 70 | 1 | 42 | 2 | ·· | ·· | ·· | ·· | ·· | ·· |
| 23 (M) | 165 | 7 | 59 | 6 | 55 | 4 | 14 | 3 | 7 | 2 |
| 24 (M) | 240 | 7 | 128 | 7 | 55 | 7 | 332 | 7 | 101 | 7 |
| 26 (M) | 66 | 1 | 127 | 3 | 80 | 2 | ·· | ·· | ·· | ·· |
| 27 (M) | 131 | 7 | 162 | 7 | 207 | 7 | 249 | 7 | ·· | ·· |
| 28 (M) | 75 | 4 | 52 | 4 | 71 | 6 | 65 | 4 | 118 | 6 |
| 29 (F) | 164 | 7/6* | 87 | 2/3/4* | ·· | ·· | ·· | ·· | ·· | ·· |
| 30 (F) | 74 | 2 | 52 | 2 | 17 | 2 | ·· | ·· | ·· | ·· |
| 32 (M) | 36 | 1 | 91 | 3 | 101 | 3 | 216 | 4 | ·· | ·· |
| 33 (F) | 30 | 1 | 80 | 1 | ·· | ·· | ·· | ·· | ·· | ·· |
|  |  |  |  |  |  |  |  |  |  |  |
| POST |  |  |  |  |  |  |  |  |  |  |
| 1 (M) | 118 | 2 | 91 | 2 | 36 | 1 | 35 | 1 | 49 | 1 |
| 2 (M) | 130 | 4 | 162 | 4 | 57 | 4 | 47 | 4 | 62 | 4 |
| 3 (F) | 77 | 3/4* | 12 | 1/2* | ·· | ·· | ·· | ·· | ·· | ·· |
| 4 (F) | 20 | 5 | 52 | 5 | 44 | 5 | 40 | 5 | ·· | ·· |
| 5 (F) | 38 | 1 | ·· | ·· | ·· | ·· | ·· | ·· | ·· | ·· |
| 6 (F) | 48 | 3 | 98 | 5 | ·· | ·· | ·· | ·· | ·· | ·· |
| 7 (F) | 85 | 4 | 100 | 4 | ·· | ·· | ·· | ·· | ·· | ·· |
| 8 (F) | 61 | 3/4* | 5 | 1/2* | 1 | 1 | ·· | ·· | ·· | ·· |
| 10 (F) | 77 | 3 | 100 | 4 | ·· | ·· | ·· | ·· | ·· | ·· |
| 11 (F) | 185 | 6 | 182 | 5 | 77 | 4 | 98 | 4 | 141 | 4 |
| 12 (F) | 99 | 3 | 6 | 1 | 177 | 7 | 264 | 7 | 1 | 1 |
| 14 (F) | 101 | 1 | 38 | 1 | 79 | 1 | ·· | ·· | ·· | ·· |
| 15 (M) | 198 | 2/2* | 143 | 2 | ·· | ·· | ·· | ·· | ·· | ·· |
| 18 (M) | 52 | 2 | 19 | 1 | 52 | 2 | 11 | 1 | ·· | ·· |
| 22 (F) | 62 | 3 | ·· | ·· | ·· | ·· | ·· | ·· | ·· | ·· |
| 23 (M) | 1 | 3 | 41 | 3 | 54 | 4 | 39 | 4 | 95 | 4 |
| 24 (M) | ·· | ·· | ·· | ·· | ·· | ·· | ·· | ·· | ·· | ·· |
| 26 (M) | 3 | 1 | 137 | 3 | 18 | 1 | 92 | 2 | 24 | 2 |
| 27 (M) | 157 | 7 | 112 | 7 | 267 | 7 | 152 | 7 | 154 | 7 |
| 28 (M) | 80 | 4 | 81 | 4 | 63 | 4 | 91 | 7 | 15 | 4 |
| 29 (F) | 25 | 2 | 16 | 2 | 92 | 4 | 11 | 2 | 7 | 2 |
| 30 (F) | 64 | 5 | 38 | 5 | 59 | 5 | 6 | 5 | ·· | ·· |
| 32 (M) | 137 | 4 | ·· | ·· | ·· | ·· | ·· | ·· | ·· | ·· |
| 33 (F) | 7 | 2 | ·· | ·· | ·· | ·· | ·· | ·· | ·· | ·· |

M, male; F, female; g, grams; BSS, Bristol Stool Score. BSS is the score for stool consistency* denotes multiple bowel movements on same day.

**Table S3:** Data are mean (± SD) characteristics at baseline and following 12-weeks of the Low Residue Diet for mtDNA-related disease patients.

|  | **Pre (n=24)** | **Post (n=24)** | **p value** |
| --- | --- | --- | --- |
| ***Dietary Recall*** |  |  |  |
| Total kcals/day | 2115 (± 986) | 1918 (± 760) | 0·50 |
| Total Carbohydrates (g/day) | 277 (± 133) | 268 (± 98) | 0·85 |
| of which sugars (g/day) | 118 (± 66) | 106 (± 56) | 0·38 |
| of which starch (g/day) | 150 (± 74) | 148 (± 59) | 0·98 |
| Total Fat (g/day) | 81 (± 40) | 69 (± 40) | 0·35 |
| of which saturates (g/day) | 29 (± 13) | 26 (± 15) | 0·46 |
| Protein (g/day) | 86 (± 41) | 71 (± 20) | 0·07 |
| Dietary Fibre (g/day) | 18 (± 8) | 12 (± 6) | 0·03* |
|  |  |  |  |
| ***Short Chain Fatty Acids*** |  |  |  |
| Acetic Acid (mmol/L) | 11 (± 9) | 7 (± 3) | 0·08 |
| Propionic Acid (mmol/L) | 3·8 (± 3·9) | 3·3 (± 1·7) | 0·61 |
| Butyrate (mmol/L) | 2·5 (± 2·4) | 1·4 (± 1·4) | 0·06 |
| IsoButyrate Acid (mmol/L) | 0·4 (± 0·8) | 0·4 (± 0·2) | 0·81 |
| Valeric (mmol/L) | 0·5 (± 0·8) | 0·4 (± 0·4) | 0·78 |
| Isovaleric (mmol/L) | 0·6 (± 0·8) | 0·8 (± 0·5) | 0·56 |
| Total SCFA (mmol/L) | 19 (± 16) | 13 (± 12) | 0·18 |
|  |  |  |  |
| ***Biochemistry*** |  |  |  |
| Ghrelin (pg/ml) | 542 (± 558) | 678 (± 835) | 0·16 |
| Leptin (pg/ml) | 17 (± 11) | 18·6 (± 12·1) | 0·30 |
| Peptide YY (pg/ml) | 172 (± 132) | 153 (± 94) | 0·40 |
| Glucagon-like peptide-2 (pg/ml) | 4·7 (0·5) | 4·6 (± 0·7) | 0·20 |
| Glucagon-like peptide-1 (pg/ml) | 164 (± 186) | 241 (± 220) | 0·01^#^ |
| Vasoactive intestinal peptide (pg/ml) | 13 (± 8) | 12 (± 6) | 0·53 |
| Nestafin.1 (pg/ml) | 4·4 (± 6·3) | 3·9 (± 3·0) | 0·65 |

**kcals, kilocalorie; g, grams; ml, millilitre; mg, micrograms; mcg, millicentigram; g, grams; mmol, millimoles; pg, pictograms. * and ^#^ denotes a significant differences at <0.01 and 0.05, respectively**

**Table S4:** Functional pathway acronyms and full names.

| **Short** | **Full** |
| --- | --- |
| **PWY 6737** | PWY_6737_starchdegradationV |
| **PWY 1042** | PWY_1042_glycolysisIV_plantcytosol_ |
| **CALVIN PWY** | CALVIN_PWY_Calvin_Benson_Basshamcycle |
| **PWY0 1319** | PWY0_1319_CDP_diacylglycerolbiosynthesisII |
| **PWY 5667** | PWY_5667_CDP_diacylglycerolbiosynthesisI |
| **PWY 6151** | PWY_6151_S_adenosyl_L_methioninecycleI |
| **PWY 5686** | PWY_5686_UMPbiosynthesis |
| **NONOXIPENT PWY** | NONOXIPENT_PWY_pentosephosphatepathway_non_oxidativebranch_ |
| **PWY 6163** | PWY_6163_chorismatebiosynthesisfrom3_dehydroquinate |
| **COMPLETE ARO** | COMPLETE_ARO_PWY_superpathwayofaromaticaminoacidbiosynthesis |
| **SER GLYSYN** | SER_GLYSYN_PWY_superpathwayofL_serineandglycinebiosynthesisI |
| **GLYCOGENSYNTH PWY** | GLYCOGENSYNTH_PWY_glycogenbiosynthesisI_fromADP_D_Glucose_ |
| **NONMEVIPP PWY** | NONMEVIPP_PWY_methylerythritolphosphatepathwayI |
| **PWY 6386** | PWY_6386_UDP_N_acetylmuramoyl_pentapeptidebiosynthesisII_lysine_containing_ |
| **PWY 6387** | PWY_6387_UDP_N_acetylmuramoyl_pentapeptidebiosynthesisI_meso_diaminopimelatecontaining_ |
| **PWY 6121** | PWY_6121_5_aminoimidazoleribonucleotidebiosynthesisI |
| **PWY 5097** | PWY_5097_L_lysinebiosynthesisVI |
| **PEPTIDOGLYCANSYN PWY** | PEPTIDOGLYCANSYN_PWY_peptidoglycanbiosynthesisI_meso_diaminopimelatecontaining_ |
| **PANTO PWY** | PANTO_PWY_phosphopantothenatebiosynthesisI |
| **PWY 6527** | PWY_6527_stachyosedegradation |
| **ANAGLYCOLYSIS PWY** | ANAGLYCOLYSIS_PWY_glycolysisIII_fromglucose_ |
| **PANTOSYN PWY** | PANTOSYN_PWY_pantothenateandcoenzymeAbiosynthesisI |
| **PWY 7199** | PWY_7199_pyrimidinedeoxyribonucleosidessalvage |
| **GALACTUROCAT PWY** | GALACTUROCAT_PWY_D_galacturonatedegradationI |
| **COA PWY** | COA_PWY_coenzymeAbiosynthesisI |
| **GALACT GLUCUROCAT** | GALACT_GLUCUROCAT_PWY_superpathwayofhexuronideandhexuronatedegradation |
| **PWY 6507** | PWY_6507_4_deoxy_L_threo_hex_4_enopyranuronatedegradation |
| **GLUCUROCAT PWY** | GLUCUROCAT_PWY_superpathwayofbeta__D_glucuronideandD_glucuronatedegradation |
| **PWY 7456** | PWY_7456_mannandegradation |
| **PWY 5177** | PWY_5177_glutaryl_CoAdegradation |
| **HEXITOLDEGSUPER PWY** | HEXITOLDEGSUPER_PWY_superpathwayofhexitoldegradation_bacteria_ |
| **PWY 6901** | PWY_6901_superpathwayofglucoseandxylosedegradation |
| **PWY 6629** | PWY_6629_superpathwayofL_tryptophanbiosynthesis |
| **PWY 6895** | PWY_6895_superpathwayofthiamindiphosphatebiosynthesisII |
| **THISYN PWY** | THISYN_PWY_superpathwayofthiamindiphosphatebiosynthesisI |
| **PWY 6628** | PWY_6628_superpathwayofL_phenylalaninebiosynthesis |
| **PWY66 409** | PWY66_409_superpathwayofpurinenucleotidesalvage |
| **ANAEROFRUCAT PWY** | ANAEROFRUCAT_PWY_homolacticfermentation |
| **PWY 5484** | PWY_5484_glycolysisII_fromfructose6_phosphate_ |
| **PWY66 400** | PWY66_400_glycolysisVI_metazoan_ |
| **GLYCOLYSIS** | GLYCOLYSIS_glycolysisI_fromglucose6_phosphate_ |

**Detailed Figure Legends**

**Figure 1:** Consort diagram of trial profile.

* Healthy matched controls attended visit 1 and 2 to report demographics and provide a 2-3g stool sample. No further outcomes were measured prior to or following visit 1 and 2 for healthy controls. BS; blood sample, BSS; Bristol Stool Score, PA; physical activity and sleep monitor, CTT; colonic transit time.

**Figure 5.** Microbiome profile for controls (n=10) and mtDNA related disease patients (n=10). A) Boxplots showing linear discriminant scores (LDA) for significantly different bacterial abundances, B) Cladogram of significantly different taxonomic differences and C) Boxplots showing LDA scores for significantly different functional profiles (functional titles can be found in Supplementary Table 1).

**Supplemental Figure Legends**

**Figure S1.** Dot plot Bristol Stool Score and stool frequency. Time points are coloured with red (baseline) and black (week 12) and the size of each point is relative to stool frequency. BSS 1-2: constipation, 3-5: normal stool and 6-7: loose stools) (n=24).

**Figure S2.** Example of Colonic Transit scan for LRD03 with 20 radiopaque markers present at pre (A) and post (B) LRD intervention.

**Figure S3.** Summary of Metagenomic Read Filtering.

**Figure S4.** Relative Contribution of RNA and Host Reads

**Figure S5.** Output of Metagenomic Reads After Filtering.

**Figure S6.** Sample size estimates. Required sample sizes for a randomised controlled trial for stool consistency. Red and blue line denote an 80 and 90% power, respectively**.**

**Figure S7.** Stacked bar plots showing the distribution of the top 10 species (A) and phylum (B) based on mean relative abundance across all samples comparing controls (n=10) mtDNA-related disease patients (n=10) pre and post the LRD intervention. Pre and post LRD intervention for patients are in the same order (i.e. position 1 is the same person in the pre and post-bar plots).

**Figure S8.** Microbiome profile for controls (n=10) and mtDNA-related disease patients (n=10). A) Boxplots for alpha diversity measures Chao1 and Shannon Diversity Index (Mann-Whitney U test of significance); B) Principle coordinate analysis (PCoA) of Weighted (I) and Unweighted (II) Unifrac distances with significance of disease status tested using PERMANOVA (~Age+BMI+Disease) and C) Boxplots showing the relative abundance of significantly different *species*. *F. prausnitzii*, *Faecalibacterium prausnitzii*; *R. intestinalis, Roseburia intestinalis; E. coli, Escherichia coli and B. bifidum, Bifidobacterium bifidum.* * denotes a significant difference between groups at baseline at p<0·05.

**Figure S9.** Pathway species contribution plots with highest relative abundance for glycolysis I (A), glycolysis VI (B) and glycolysis II (C) comparing controls (n=10) and mtDNA-related disease patient (n=10) pre and post the LRD intervention.

**Figure S10.** Pathway species contribution plots for tryptophan (A) and phenylalanine biosynthesis (B) comparing controls (n=10) and mtDNA-related disease patients pre and post LRD (n=10) pre and post the LRD intervention.

**Figure S11**. Microbiome profile for mtDNA related disease patients pre (n=10) and post (n=10) the LRD intervention. A) Boxplots for alpha diversity measures Chao1 and Shannon Diversity Index; B) Principle coordinate analysis (PCoA) of Weighted (I) and Unweighted (II) Unifrac Analysis and C) Boxplots showing the relative abundance of *species* that were significantly different between healthy controls and mtDNA disease patients. *F. prausnitzii*, *Faecalibacterium Prausnitzii*; *R. intestinalis, Roseburia intestinalis; E. coli, Escherichia coli and B. bifidum, Bifidobacterium bifidum*.

**Figure S12.** Boxplots showing linear discriminant scores (LDA) for significantly different bacterial relative abundances comparing controls (n=10) and mtDNA-related disease patient pre and post LRD (n=10).

**Figure S13.** Boxplots showing comparing Weighted and Unweighted UniFrac beta diversity distance comparing controls (n=10) and mtDNA-related disease patient pre and post LRD (n=10).

**Figure S14.** Scatterplot of pairwise correlations between gut microbiome and questionnaire data relating to gastrointestinal (GI) symptoms (Neurological Bowel Dysfunction (NBD) score, Patients Assessment of Constipation Symptoms (PAC-SYM), ROME III and stool frequency and consistency (SC)) at A - baseline (n-10) and B - 12-weeks (n=10) following the LRD.


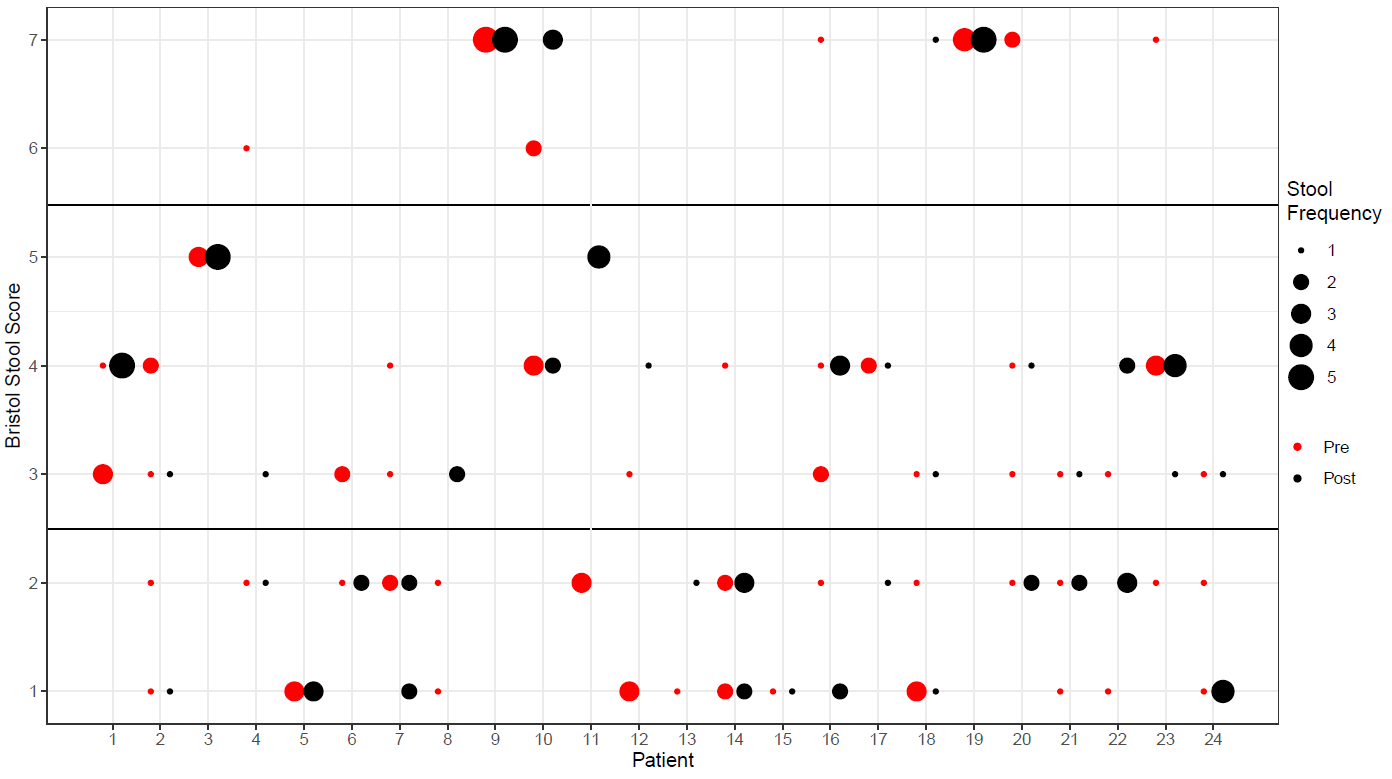


## Figure S1.


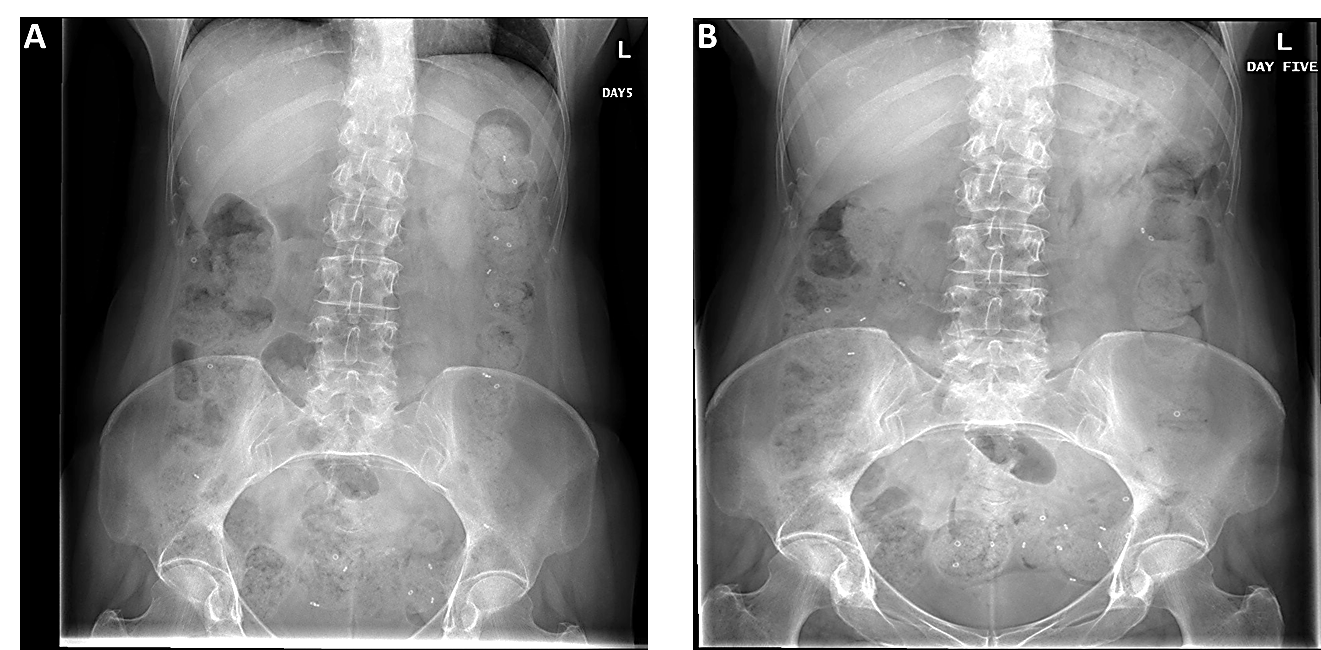


**Figure S2.**


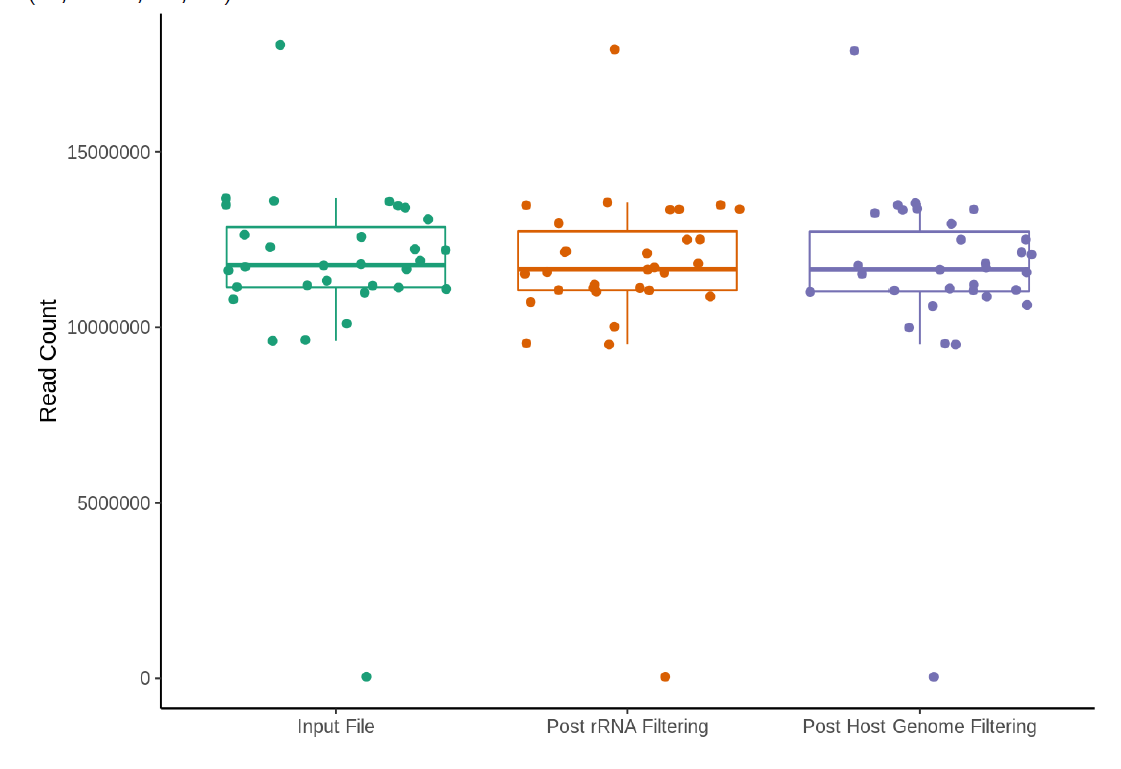


**Figure S3.**


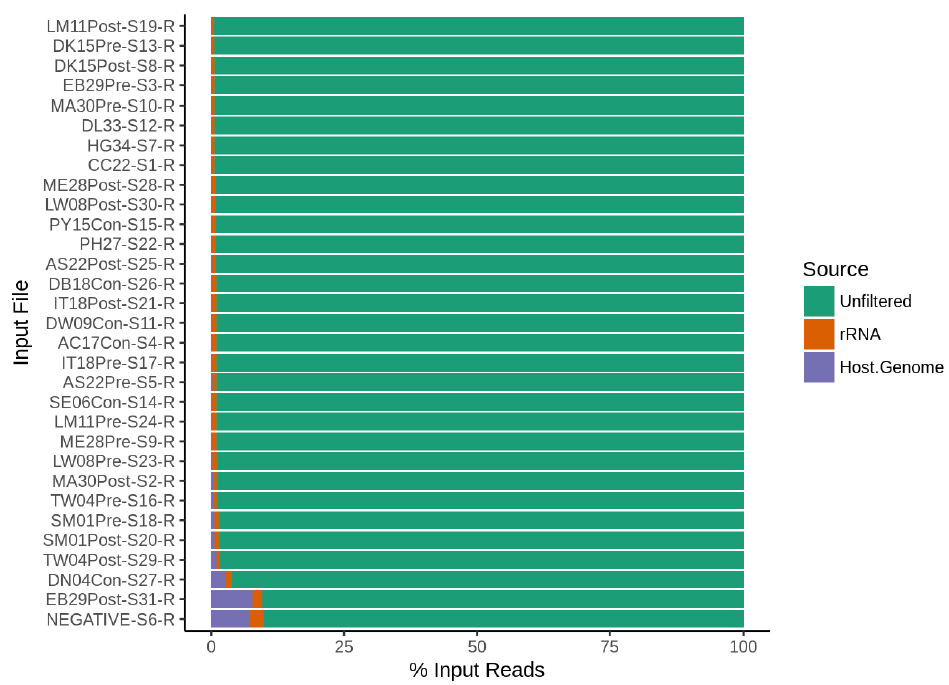


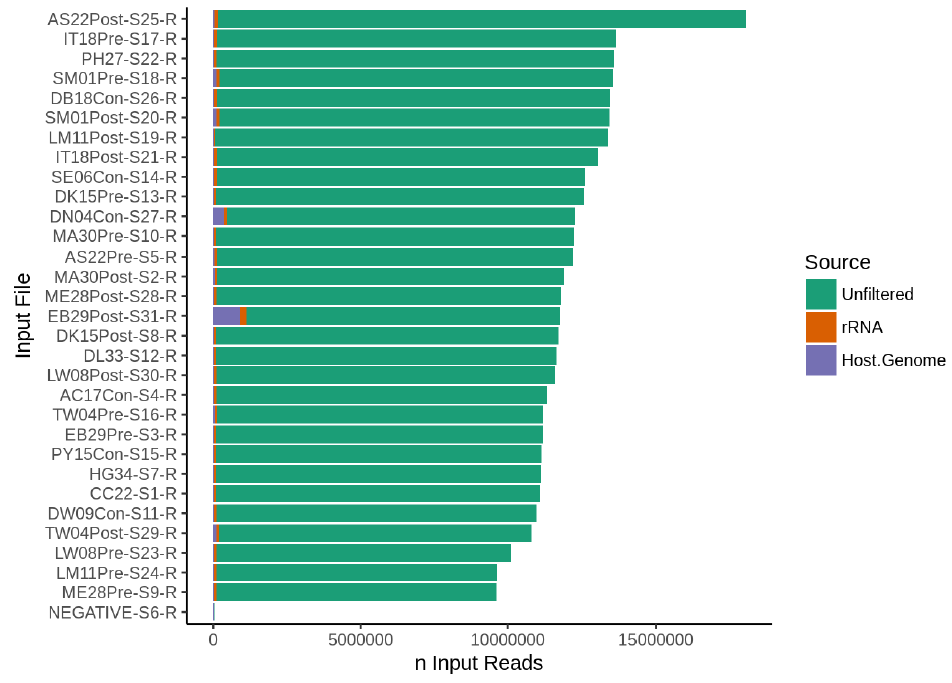


**Figure S4.**


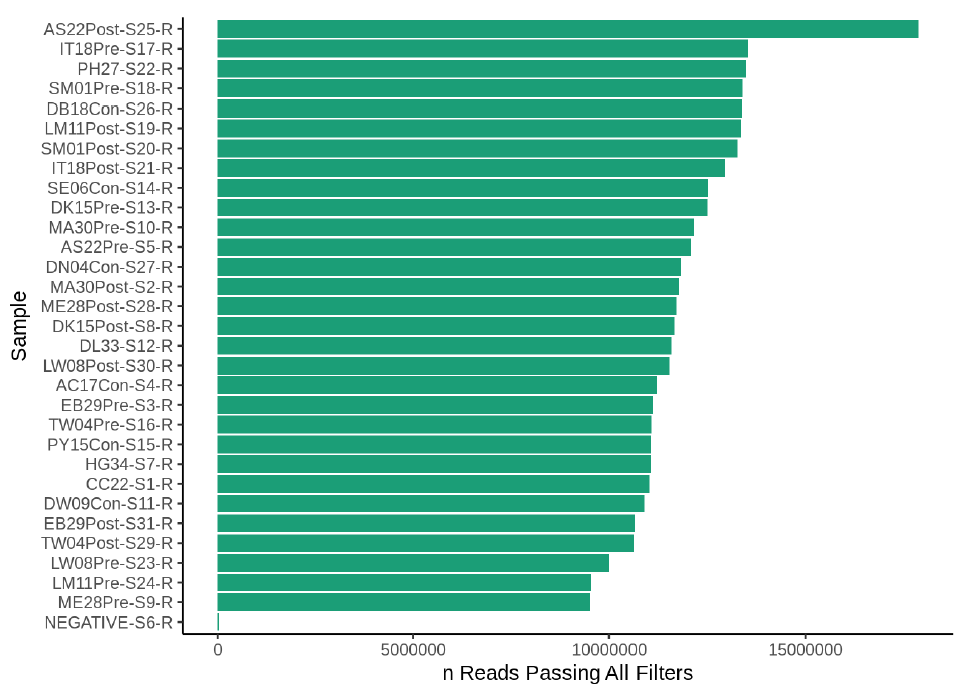


**Figure S5.**


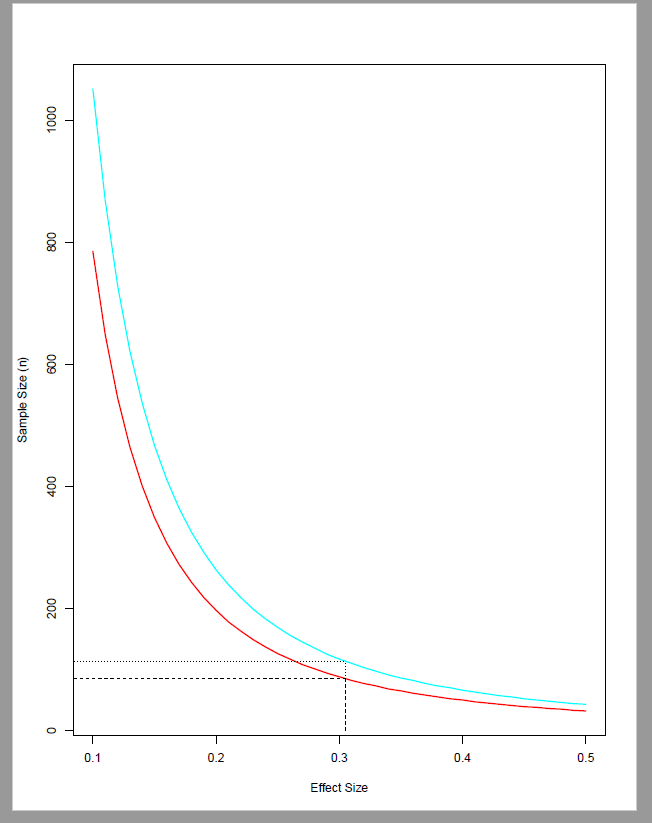


**Figure S6..**

**
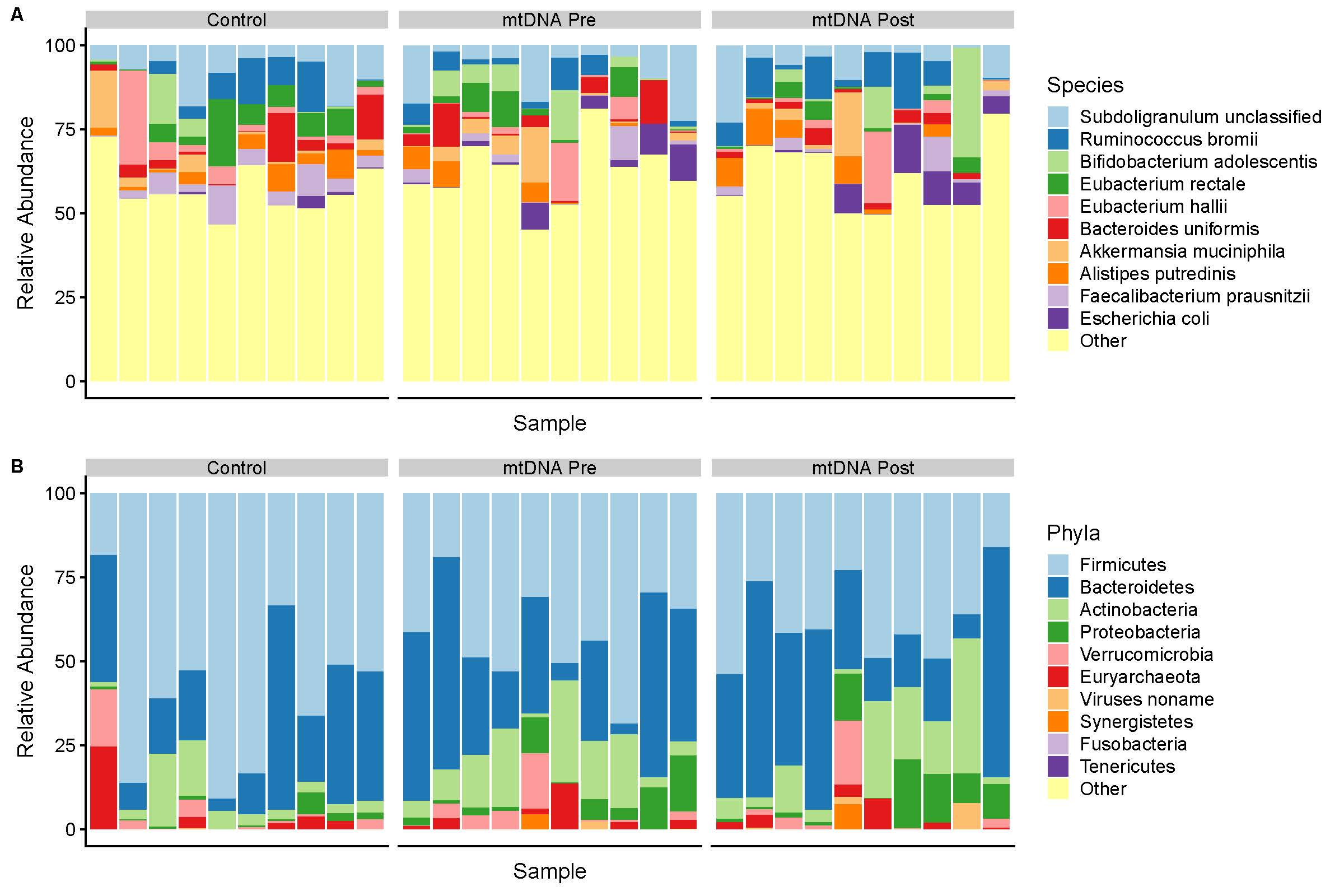
**

**Figure S7.**

**
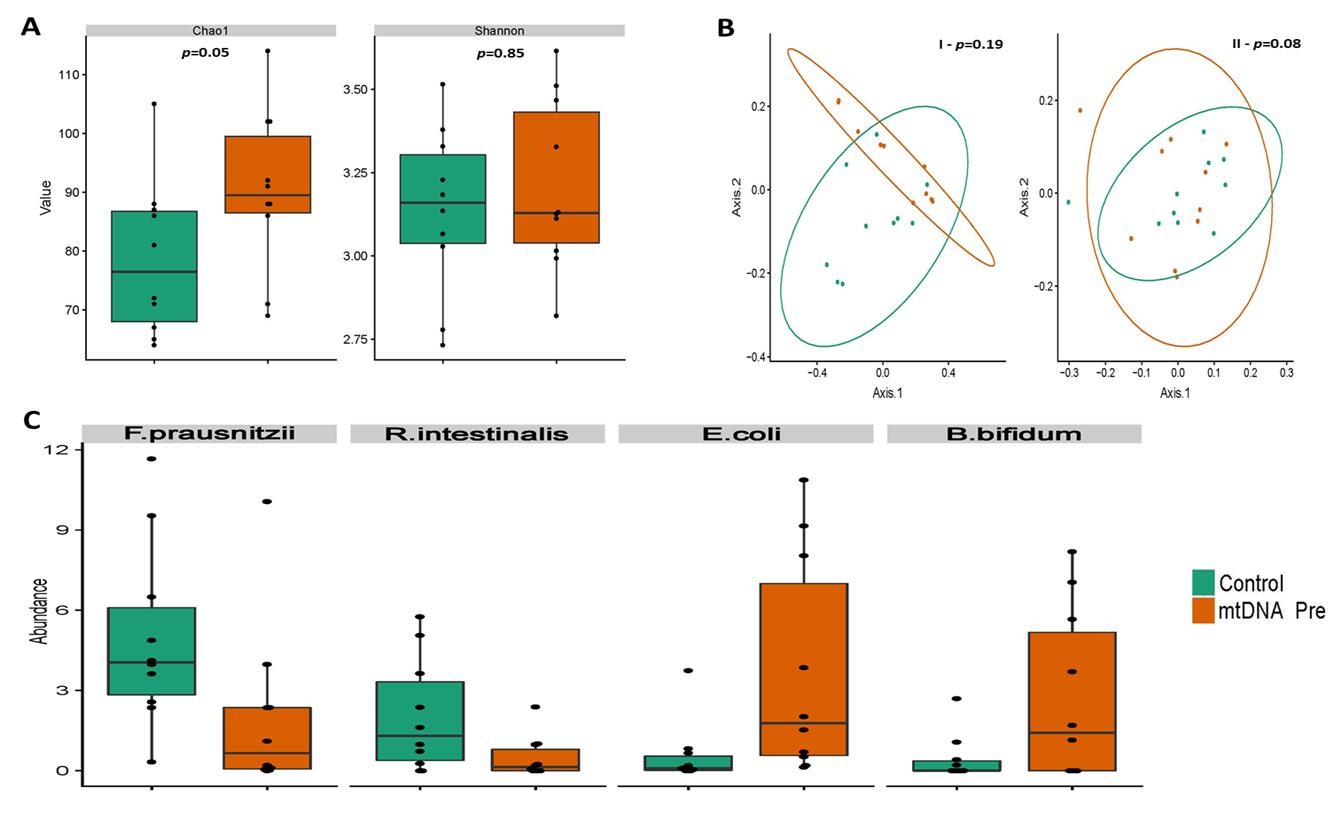
**

**Figure S8.**

**
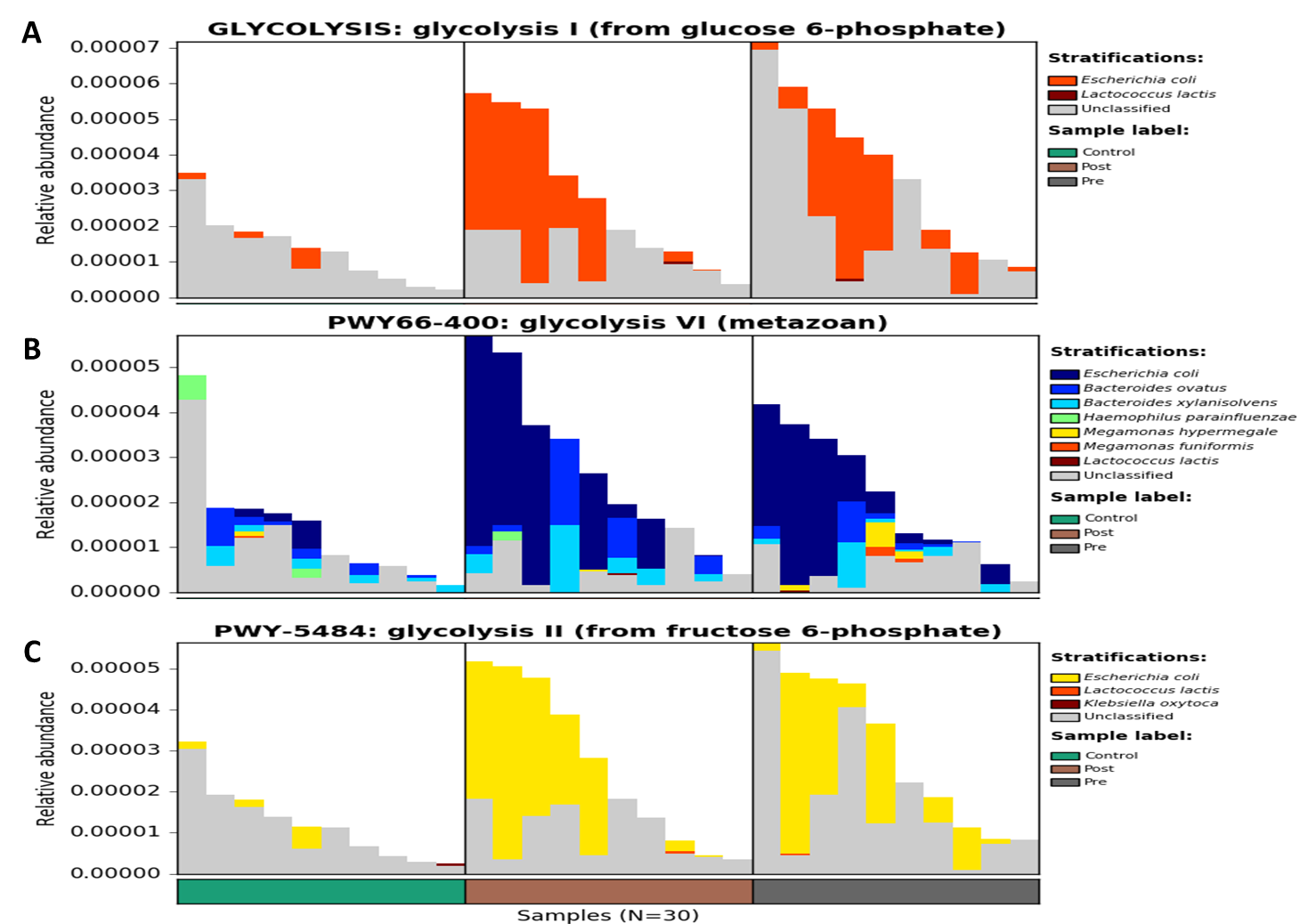
**

**Figure S9.**

**
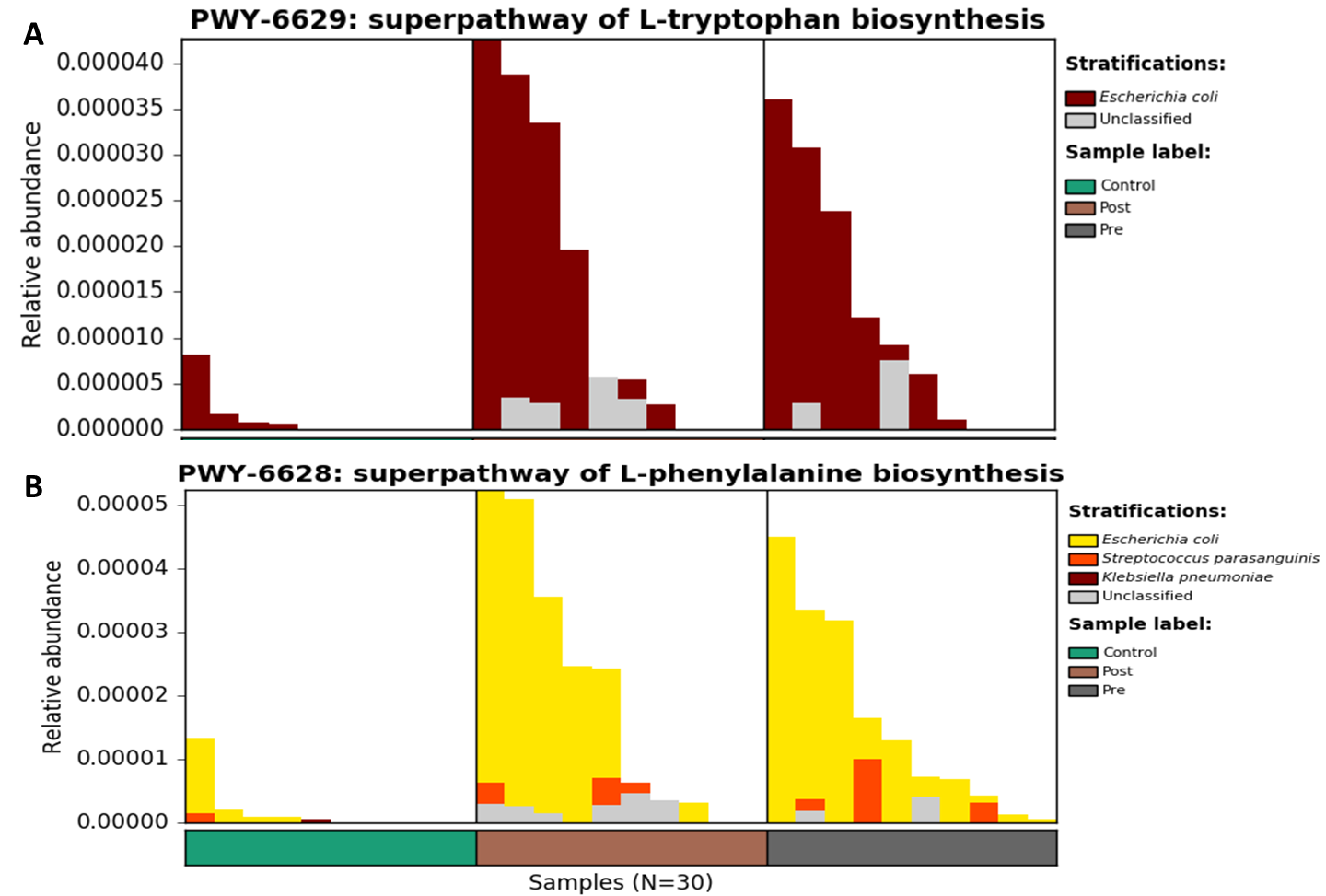
**

**Figure S10.**

**
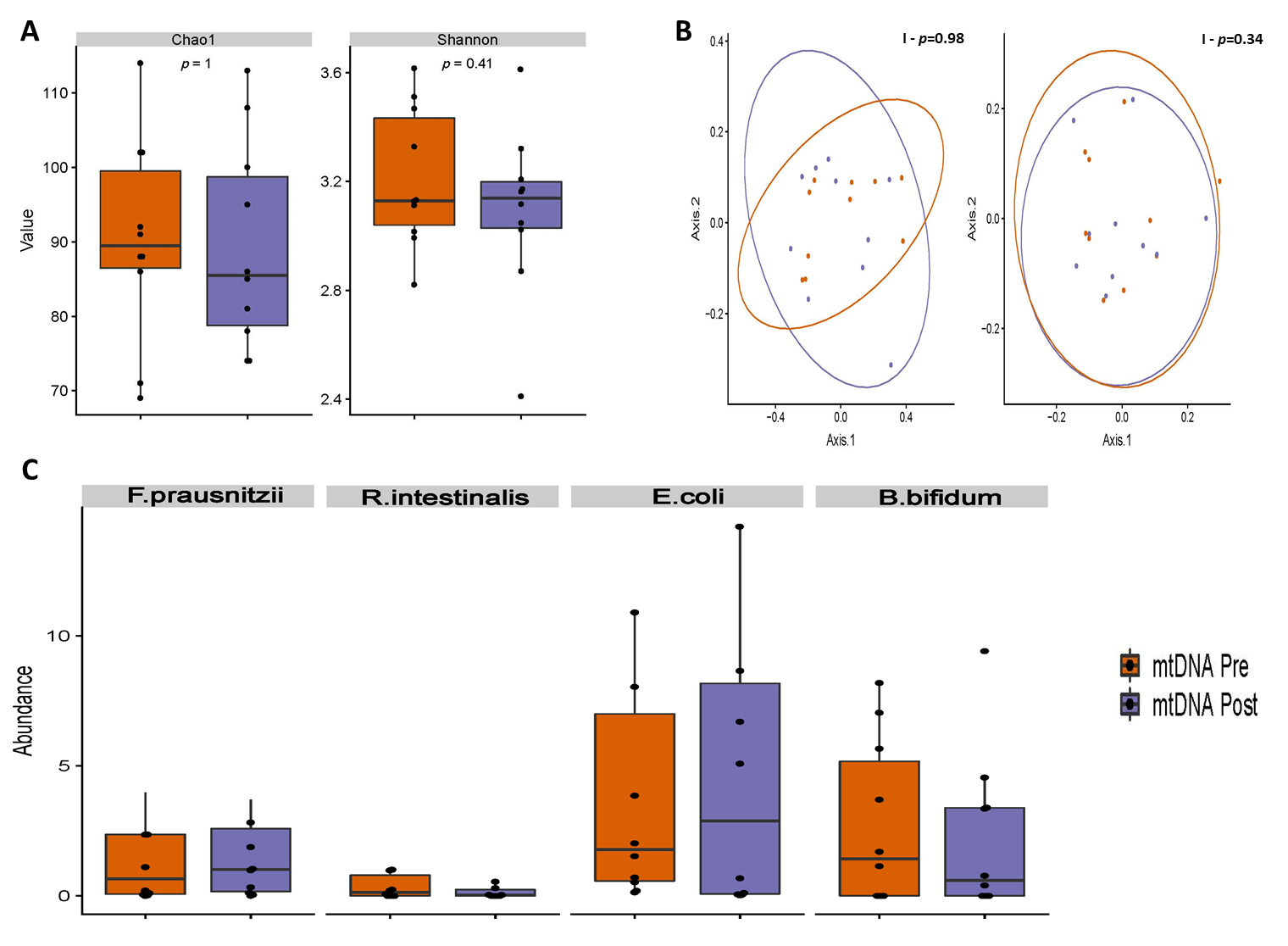
**

**Figure S11**.

**
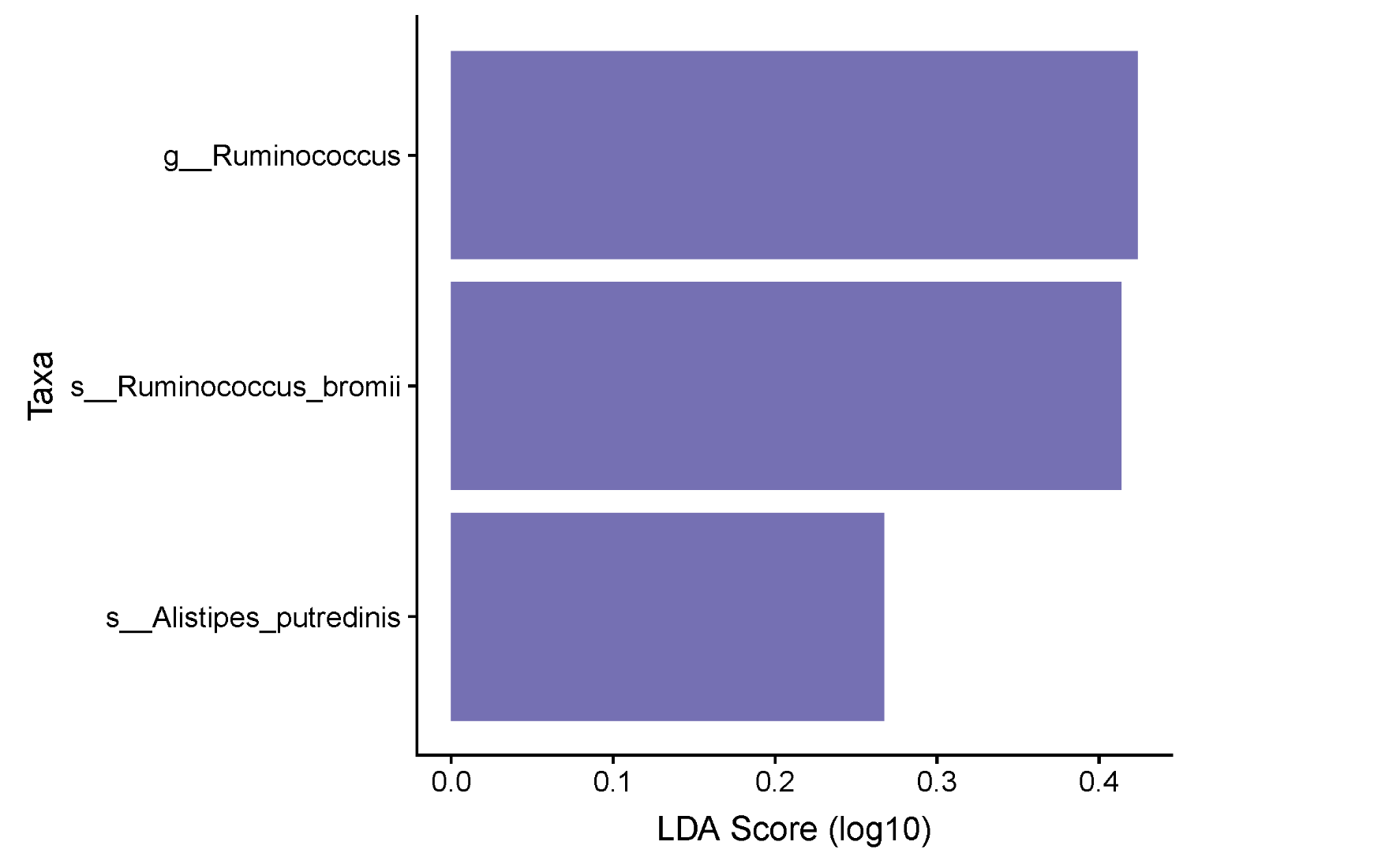
**

**Figure S12**.

**
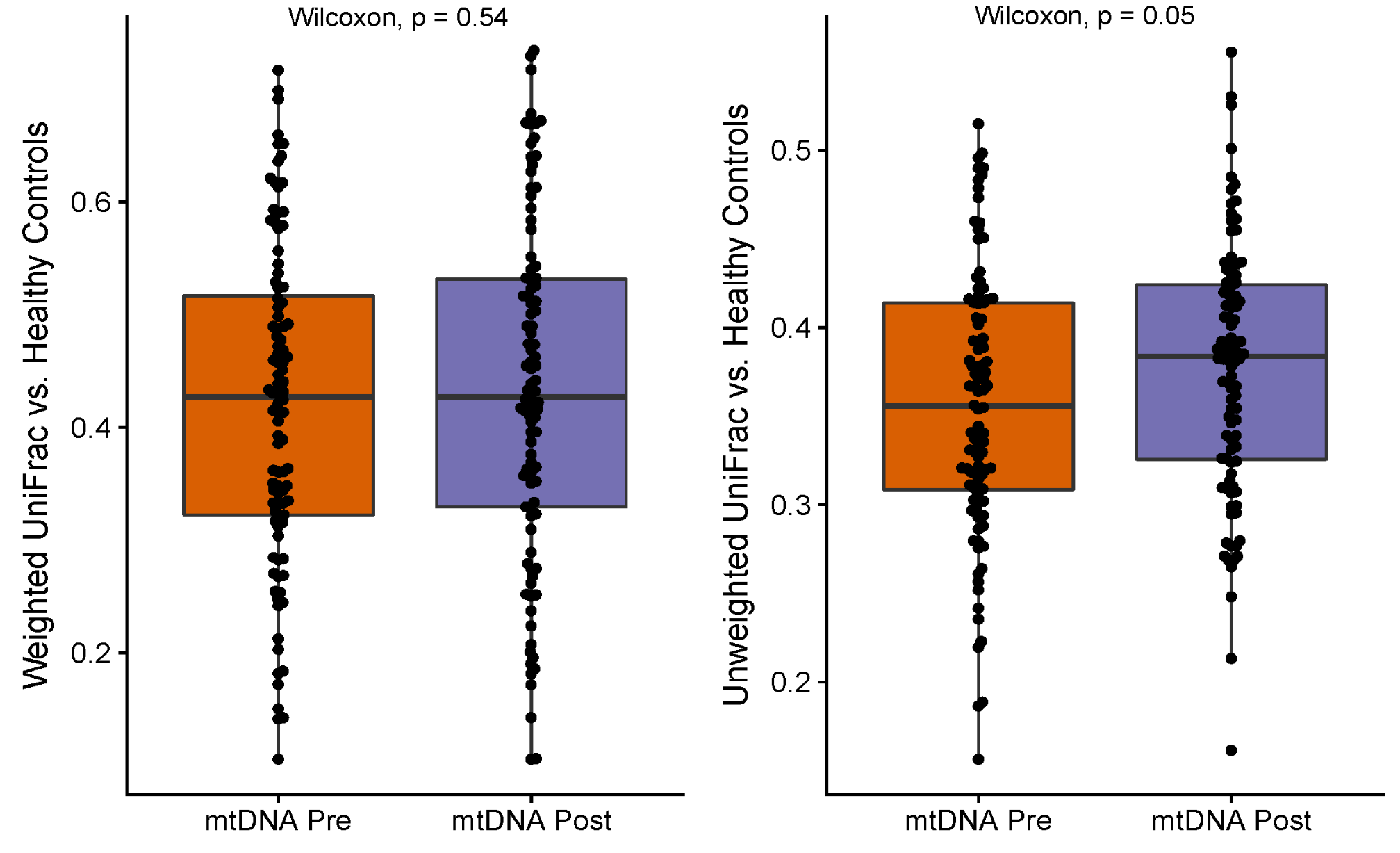
**

**Figure S13.**


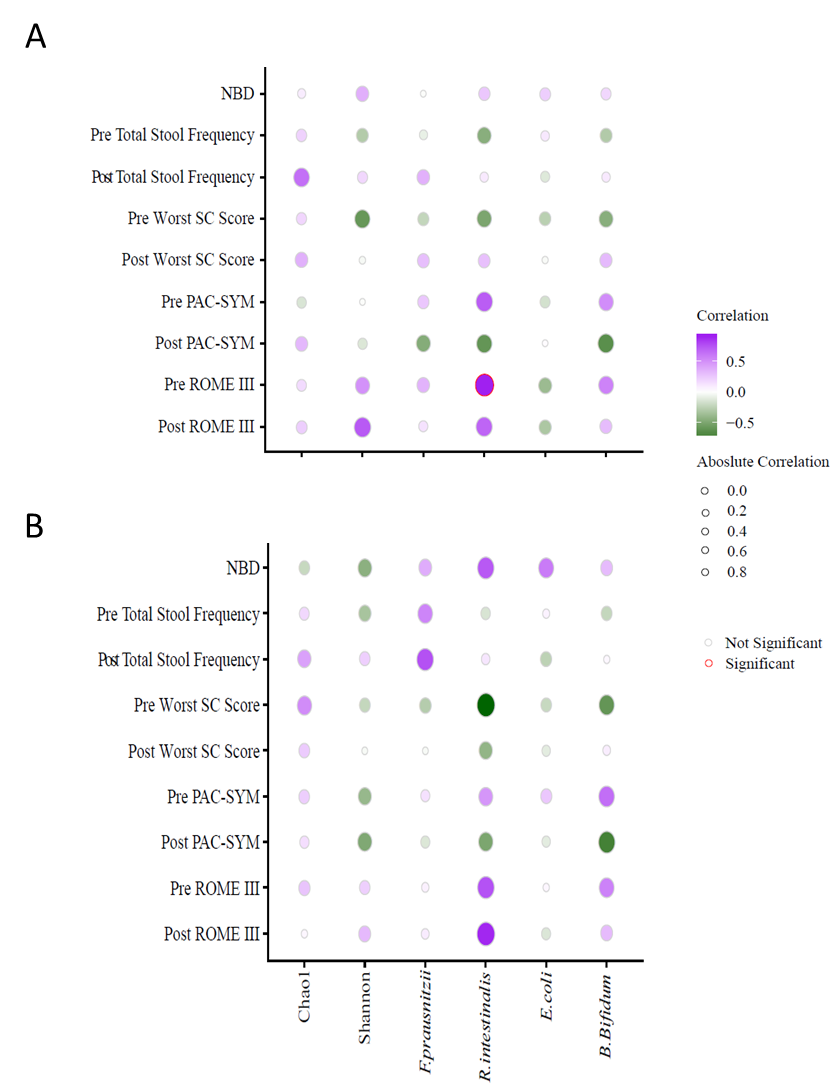


**Figure S14.**

**References**

1. Rao SS, Camilleri M, Hasler WL, Maurer AH, Parkman HP, Saad R, et al. Evaluation of gastrointestinal transit in clinical practice: position paper of the American and European Neurogastroenterology and Motility Societies. Neurogastroenterol Motil. 2011;23(1):8-23.

2. Evans RC, Kamm MA, Hinton JM, Lennard-Jones JE. The normal range and a simple diagram for recording whole gut transit time. Int J Colorectal Dis. 1992;7(1):15-7.

3. Heaton KW, Radvan J, Cripps H, Mountford RA, Braddon FE, Hughes AO. Defecation frequency and timing, and stool form in the general population: a prospective study. Gut. 1992;33(6):818-24.

4. Thompson WG, Heaton KW. Irritable Bowel Syndrome. 2nd ed. Abingdon: Health Press; 2003.

5. Frank L, Kleinman L, Farup C, Taylor L, Miner P, Jr. Psychometric validation of a constipation symptom assessment questionnaire. Scandinavian journal of gastroenterology. 1999;34(9):870-7.

6. de Vries PR, Janssen M, Spaans E, de Groot I, Janssen A, Smeitink J, et al. Natural variability of daily physical activity measured by accelerometry in children with a mitochondrial disease. Mitochondrion. 2019;47:30-7.

7. Simpson E, Bradley J, Poliakov I, Jackson D, Olivier P, Adamson AJ, et al. Iterative Development of an Online Dietary Recall Tool: INTAKE24. Nutrients. 2017;9(2).

8. Mathers JC, Tagny JM. Diurnal changes in large-bowel metabolism: short-chain fatty acids and transit time in rats fed on wheat bran. The British journal of nutrition. 1994;71(2):209-22.

9. Houghton D, Stewart CJ, Stamp C, Nelson A, Aj Ami NJ, Petrosino JF, et al. Impact of Age-Related Mitochondrial Dysfunction and Exercise on Intestinal Microbiota Composition. J Gerontol A Biol Sci Med Sci. 2018;73(5):571-8.

10. Truong DT, Franzosa EA, Tickle TL, Scholz M, Weingart G, Pasolli E, et al. MetaPhlAn2 for enhanced metagenomic taxonomic profiling. Nat Methods. 2015;12(10):902-3.

11. Franzosa EA, McIver LJ, Rahnavard G, Thompson LR, Schirmer M, Weingart G, et al. Species-level functional profiling of metagenomes and metatranscriptomes. Nat Methods. 2018;15(11):962-8.

12. McMurdie PJ, Holmes S. phyloseq: an R package for reproducible interactive analysis and graphics of microbiome census data. PloS one. 2013;8(4):e61217.

13. R Development Core Team. R: A language and environment for statistical computing. Vienna, Austria: R Foundation for Statistical Computing; 2010.

14. Pasolli E, Schiffer L, Manghi P, Renson A, Obenchain V, Truong DT, et al. Accessible, curated metagenomic data through ExperimentHub. Nat Methods. 2017;14(11):1023-4.

15. Oksanen J, Blanchet FG, Kindt R, Legendre P, Minchin PR, O’Hara RB, et al. Vegan: community ecology package. R package vegan, vers. 2.2-1. 2015.

16. Mandal S, Van Treuren W, White RA, Eggesbo M, Knight R, Peddada SD. Analysis of composition of microbiomes: a novel method for studying microbial composition. Microb Ecol Health Dis. 2015;26:27663.

17. Segata N, Izard J, Waldron L, Gevers D, Miropolsky L, Garrett WS, et al. Metagenomic biomarker discovery and explanation. Genome Biol. 2011;12(6):R60.

18. Rice P, Longden I, Bleasby A. EMBOSS: the European Molecular Biology Open Software Suite. Trends Genet. 2000;16(6):276-7.
